# A Universal Strategy for Enhancing the Circulating miRNAs’ Detection Performance of Rolling Circle Amplification by Using a Dual-Terminal Stem-Loop Padlock

**DOI:** 10.1021/acsnano.3c07721

**Published:** 2023-12-27

**Authors:** Hanqing Xu, Xianlan Wu, Qian Liu, Cheng Yang, Man Shen, Yingran Wang, Shuai Liu, Shuang Zhao, Ting Xiao, Minghui Sun, Zishan Ding, Jing Bao, Ming Chen, Mingxuan Gao

**Affiliations:** § Department of Clinical Laboratory Medicine, Southwest Hospital, Third Military Medical University (Army Medical University), Chongqing 400038, P. R. China; ‡ College of Pharmacy and Laboratory Medicine, Third Military Medical University (Army Medical University), 30 Gaotanyan, Shapingba District, Chongqing 400038, P. R. China

**Keywords:** Rolling Circle Amplification, Padlock, Structural Reconstruction, Stem-Loop, Circulating miRNAs

## Abstract

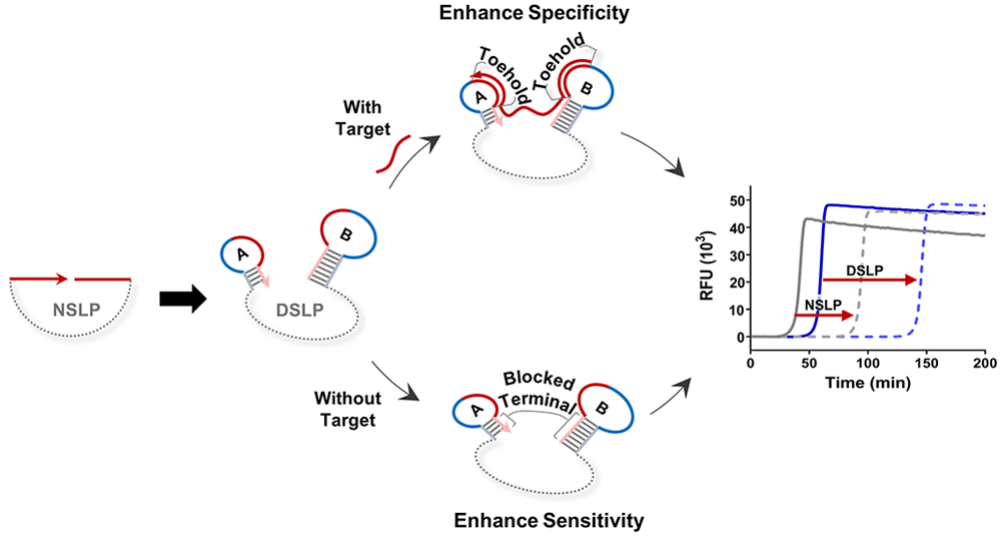

Rolling circle amplification (RCA) is one of the most promising nucleic acid detection technologies and has been widely used in the molecular diagnosis of disease. Padlock probes are often used to form circular templates, which are the core of RCA. However, RCA often suffers from insufficient specificity and sensitivity. Here we report a reconstruction strategy for conventional padlock probes to promote their overall performance in nucleic acid detection while maintaining probe functions uncompromised. When two rationally designed stem-loops were strategically placed at the two terminals of linear padlock probes, the specificity of target recognition was enhanced and the negative signal was significantly delayed. Our design achieved the best single-base discrimination compared with other structures and over a 1000-fold higher sensitivity than that of the conventional padlock probe, validating the effectiveness of this reconstruction. In addition, the underlying mechanisms of our design were elucidated through molecular dynamics simulations, and the versatility was validated with longer and shorter padlocks targeting the same target, as well as five additional targets (four miRNAs and dengue virus – 2 RNA mimic (DENV-2)). Finally, clinical applicability in multiplex detection was demonstrated by testing real plasma samples. Our exploration of the structures of nucleic acids provided another perspective for developing high-performance detection systems, improving the efficacy of practical detection strategies, and advancing clinical diagnostic research.

## Introduction

Compared to traditional protein biomarkers, nucleic acid biomarkers can respond to the disease state at the transcriptional level, which is the most promising field for early precision diagnosis and treatment.^1,2^ Rolling circle amplification (RCA) is one of the most promising isothermal amplification techniques and has been used in a wide range of fields, such as nanomaterials, biochemistry, and molecular biology.^3−5^ In nucleic acid detection, padlock probes are commonly used to specifically identify nucleic acid targets and be connected into circular templates, which is then followed by RCA for signal amplification and output, which is commonly involved in molecular diagnosis of various diseases.^6,7^ Conventional padlock probes are linear DNA sequences that typically contain two target recognition regions at both terminals and a functional region in the middle. By changing the target recognition regions, padlock probes can target different nucleic acid molecules.^8^ Moreover, by rationally designing the functional region, RCA products can output signals in various ways, such as by forming G quadruplexes, combining with hemin, thioflavin, or zinc phthalocyanine to catalyze the color development of substrates, or forming photoelectric signals,^9,10^ triggering hyperbranched RCA (HRCA), and outputting fluorescence signals through the intercalation of fluorescent dyes.^11^

Numerous studies have achieved effective nucleic acid detection using padlock-assisted RCA because of its advantages of efficient isothermal amplification, easy editing, and flexible signal output. However, for nucleic acid molecules with low abundance and high intermolecular homology, such as circulating miRNAs,^12,13^ the conventional padlock still has some shortcomings. For instance, the target recognition process only depends on base complementary pairing and the fidelity of the utilized ligase to ensure specificity, which is insufficient for distinguishing single-base mismatch.^14^ Additionally, nonspecific cyclization and intermolecular cross-reaction can occur,^15^ which significantly reduces the signal-to-noise ratio (SNR) and limits the detection sensitivity.

To meet the high demands of nucleic acid detection, extensive research has been devoted to improving the assay performance. The combination of several amplification strategies to achieve multiplex amplification,^16^ the combination with sensitizing nanomaterials to achieve signal amplification,^17^ and the combination with ultrasensitive sensor platforms for biosensing have all effectively improved the detection sensitivity.^18^ The selection of higher-fidelity enzymes and toehold-based strand replacement initiation also significantly improved the specificity.^19,20^ However, the most relevant strategies focused on the introduction of efficient amplification methods or sensitive detecting platforms but ignored the rational design of the padlock structure itself to obtain optimal performance or failed to improve sensitivity and specificity simultaneously. Moreover, some of them suffer from complex designs, poor versatility, difficulties in material synthesis, or lack of stable assay platforms, making them difficult to promote and apply in clinical practice.^21,22^

Therefore, we sought to explore the possibility of using the structural reconstruction strategy to improve both the specificity and sensitivity of padlock-assisted RCA. Previous studies demonstrated that manipulating the structure of the oligonucleotides is an effective method to ameliorate performance. For instance, Rossetti et al. employed hairpin DNA sequences as fluorescence-resonance-energy-transfer (FRET)-based reporters for the CRISPR-Cas system instead of linear single-stranded DNA sequences, resulting in a higher affinity with Cas12a enzymes and an enhanced trans-cleavage activity.^23^ In addition, Cui and co-workers indicated that the introduction of a hairpin structure near either the 3′ or 5′ terminal of a linear oligonucleotide is an effective way of suppressing intermolecular cross-reactions and improving the efficiency of monomeric formation.^24^ All of these reports indicated that the introduction of secondary structure elements such as hairpins into a linear template significantly influences the overall efficiency of the reaction. This hints at the possibility of improving the detection performance of the RCA by reconstructing the structure of the conventional linear padlock probe while maintaining the functionality of other regions.

In this work, we reconstructed a conventional linear padlock probe (non-stem-loop padlock, NSLP) and significantly improved the detection performance of RCA. By comparing various padlock probe structures, we experimentally and theoretically verified that the dual-terminal stem-loop padlock (DSLP) design simultaneously improved the specificity and sensitivity of RCA. The versatility of the design was then validated using longer and shorter padlocks targeting the same target, as well as five additional targets. Furthermore, we achieved multiplex detection of plasma samples from cancer patients, demonstrating the clinical practicability of our design. We believe that using a simplified design strategy to realize a more sensitive and accurate clinical test technique will have a superior effect on the application in clinical practice.

## Results and Discussion

### Principle and Feasibility of the DSLP Design

**Scheme 1:**
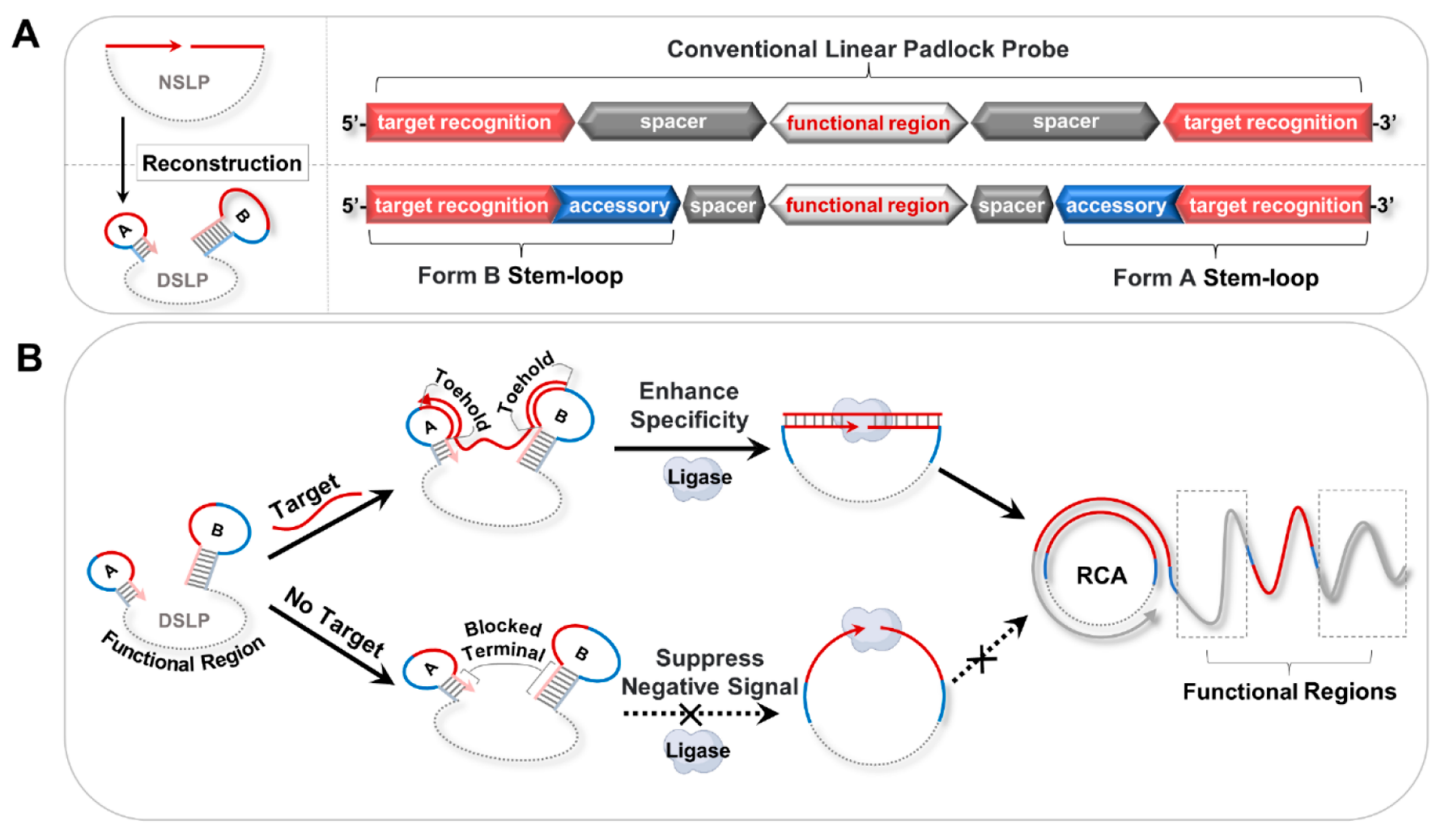
Illustration of the Padlock Probe Reconstruction Strategy. (A) Schematic of the Reconstruction from the Conventional NSLP Probe to the DSLP Probe. (B) Principle of the DSLP Design to Enhance Detection Performance

A conventional NSLP probe consists of two target recognition regions (at both terminals), one functional region (in the middle), and two spacers between them. To improve the RCA detection performance under the premise of retaining the functional region of the padlock, we introduced the DSLP design strategy (Scheme 1A). Taking miR-10b as the model target, we reconstructed both terminals of a conventional NSLP into two individual stem-loops A and B (SL-A and SL-B) by appending two accessory sequences. SL-A and SL-B blocked the 3′ and 5′ terminals of the padlock in stems, respectively. When the target presents, it must hybridize with both toeholds of the loops first and then open the stems through entropy-driven strand displacement. The target eventually pulls the two terminals of the padlock together and ligates them to form a ring. The toehold-assisted padlock strategy integrates the advantages of both toehold-mediated strand displacement and padlock-based cyclization to effectively enhance the specificity of target recognition. Meanwhile, in the absence of the target, both terminals of the padlock are blocked in stems, inhibiting nonspecific ring formation and suppressing negative signals (Scheme 1B). Furthermore, the presence of terminal stem-loops may suppress intermolecular cross-reaction due to both thermodynamic and kinetic factors to further improve the SNR.^24^ Theoretically, our reconstruction strategy could compensate for the shortcomings of padlocks.

We first examined the effect of reconstruction on the padlock function to verify the feasibility of the DSLP design. Denatured polyacrylamide gel electrophoresis (PAGE) was used to characterize the reaction process of DSLP, which showed that the reconstructed padlock could be efficiently ligated (lanes 3 and 5) and achieved RCA (lane 7) and HRCA (lane 9) only if the target existed (Figure S1). In this work, the functional region was designed as a C-rich sequence. Linear G-rich sequences amplified from RCA were used as G-quadruplex catalytic units and primer sites of HRCA, respectively, to further examine the effectiveness of the functional region on the padlock. When the target was present, a chromogenic reaction and the expected response to different concentrations of the target were achieved with a 4.4-fold absorption increase at 370 nm (Figures S2 and S3), and significantly increased HRCA fluorescence was observed (Figure S4). All of these results indicated that the reconstruction on the conventional linear padlock did not affect the functional region of the padlock.

**Figure 1:**
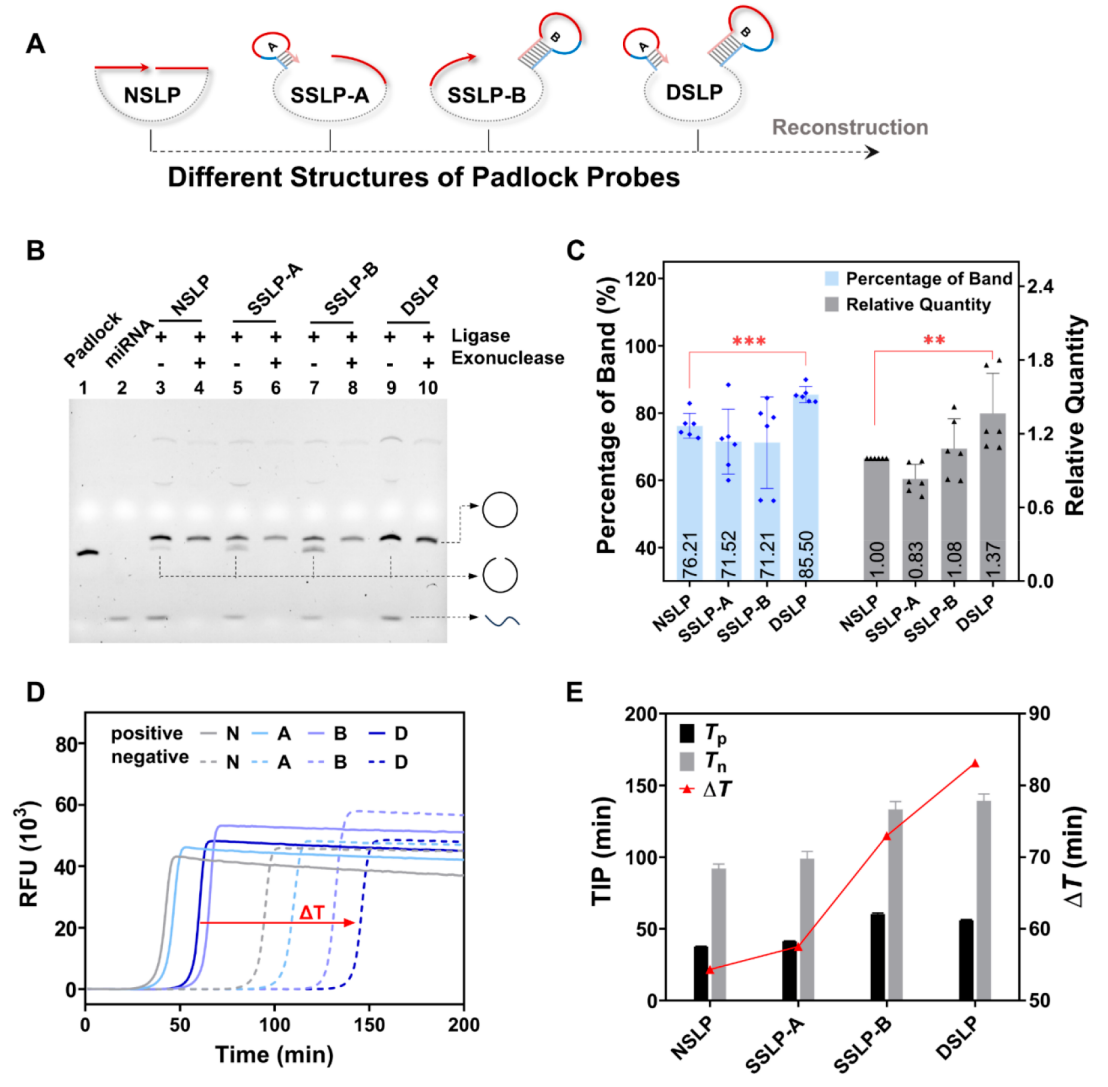
Feasibility verification of reconstruction. (A) Schematic of a conventional NSLP probe reconstructed into different structures. NSLP: without stem-loop; SSLP-A (single-terminal stem-loop padlock A): with one stem-loop at the 3′ terminal; SSLP-B (single-terminal stem-loop padlock B): with one stem-loop at the 5′ terminal; DSLP: with two stem-loops at both terminals. (B) Gel electrophoresis patterns with or without exonucleases I and III to digest noncyclic products. *C*_padlock_ = 500 nM, *C*_miRNA_ = 500 nM, *C*_PBCV-1 DNA ligase_ = 1.25 U/μL, 1X buffer, 25 °C, 3 h. (C) Quantitative analysis of band intensities in (B). Left (blue): percentages of monomeric loop bands in lanes 3, 5, 7, and 9; right (gray): relative intensities of monomeric loop bands in lanes 6, 8, and 10 compared with that of lane 4 (*n* = 6). NSLP and DSLP were compared using a *t* test (two-tailed) in the percentage of band and a Mann–Whitney test (two-tailed) in relative quantity. *** indicates statistical significance (*P* < 0.001), ** indicates statistical significance (*P* < 0.01). (D) HRCA fluorescence amplification profiles of the cyclization products of four padlock probe structures with and without the target. N, A, B, and D stand for NSLP, SSLP-A, SSLP-B, and DSLP, respectively. (E) Quantitative analysis of the difference between the positive signal (with target) and negative signal (without target) in (D), calculated from the TIP of the fluorescence amplification profiles. Mean ± SD, *n* = 3. *T*_p_: TIP of the positive signal (with target); *T*_n_: TIP of the negative signal (without target).

Subsequently, we compared different structures of padlock probes (Figure 1A, NSLP: without stem-loop; SSLP-A: with one stem-loop at the 3′ terminal; SSLP-B: with one stem-loop at the 5′ terminal; DSLP: with two stem-loops at both terminals) in terms of their efficiency in cyclizing into a monomeric loop and the suppression effect on negative signals. We compared the cyclization reaction between NSLP and DSLP (Figure S5A),^25^ which confirmed the DSLP’s superiority in promoting monomer cyclization when compared to NSLP. The PAGE results at distinct time points (T_1_/T_2_/T_3_) corresponded to the fluorescent amplification signal (Figure S5B), which indicated that the involvement of DSLP predominantly leads to delayed negative signals in comparison to NSLP.

After the priority of DSLP over NSLP was confirmed, ligation reactions of four kinds of padlock probes were conducted under the same conditions. The positive products before and after digestion were analyzed using denatured PAGE, and DSLP showed the strongest intensity of the monomeric loop bands (Figure 1B). Then, we quantified the band intensities in PAGE. DSLP demonstrated the highest monomer cyclization efficiency among those four structures, as evidenced by a 12% increase in the percentage of monomer rings before digestion (Figure 1C, blue) and a 37% increase in relative quantification after digestion (Figure 1C, gray) compared with those of the NSLP. Theoretically, terminal secondary structures are not conducive to the cyclization of the padlock probe by the target. The phenomenon we obtained may have been due to the inhibition of intermolecular cross-reactions by the terminal stem-loops, which increased the valid cyclization.

HRCA fluorescence amplification profiles of the four studied padlock probe structures with and without target are shown in Figure 1D. The time of inflection point (TIP) of the amplification curves was recorded and used as a quantitative indicator.^26^ As shown by the different colored solid curves on the left of Figure 1D, the presence of stem-loops had a limited effect on the TIP of positive signals (*T*_p_) with a slight extension from 38 min (NSLP) to 56 min (DSLP). However, as shown by the dashed curves on the right side, the increase in the stem-loop number dramatically delayed the negative signals (*T*_n_) from 92 min (NSLP) to 139 min (DSLP), and the DSLP got the largest TIP difference (Δ*T* = 83 min) between positive and negative signals among all the padlock types. A quantitative analysis of the amplification curves (*n* = 3) also gave consistent results (Figure 1E). Notably, the positive signals of HRCA were not consistent with the valid cyclization in Figure 1C, which was caused by the stem-loop structure in the circular templates that reduced the rate of HRCA amplification. We believe that the DSLP that eventually obtained the largest Δ*T* was aroused by two aspects: First, the presence of terminal stem-loops increased the valid cyclization. Therefore, positive signals were less delayed, although the stem-loop slightly reduced the rate of HRCA amplification. Second, the stem-loop could have spatially blocked the interaction between the padlock probes and the ligase, which inhibited the formation of ring structures in the absence of target sequences.

### Optimization of Experimental Conditions and Padlock Structure

We optimized the main experimental parameters to achieve the best RCA detection performance. The concentration of the buffer and the padlock probe, as well as the cyclization temperature and time, were systematically varied, and the TIP was used to quantify the signal of HRCA. (*T*_n_ – *T*_p_)/*T*_p_ and (*T*_MM_ – *T*_PM_)/*T*_PM_ were used to indicate sensitivity and specificity, respectively (*T*_MM_: the TIP of the mismatched target signal; *T*_PM_: the TIP of the perfectly matched target signal). We obtained optimal detection performance at a buffer concentration of 0.05X (Figure S6), a padlock probe concentration of 25 nM (Figure S7), a temperature of 25 °C (Figure S8), and a cyclization time of 15 min. When (*T*_n_ – *T*_p_)/*T*_p_ was used as an optimization parameter, we seemed to have obtained the optimal cyclization time at 60 min (Figure S9A). However, with a reaction time of 60 min, the detection system showed poor specificity (Figure S9B). Further analysis of Figure S9A revealed that the difference in TIP of the negative signals within 60 min was not statistically significant, nor was the difference in the positive signals, which meant that the cyclization times of 15, 30, and 60 min had no significant effect on sensitivity. On the basis of the above analysis, we finally chose 15 min as the optimal time with (*T*_MM_ – *T*_PM_)/*T*_PM_ as the optimization parameter.

**Figure 2:**
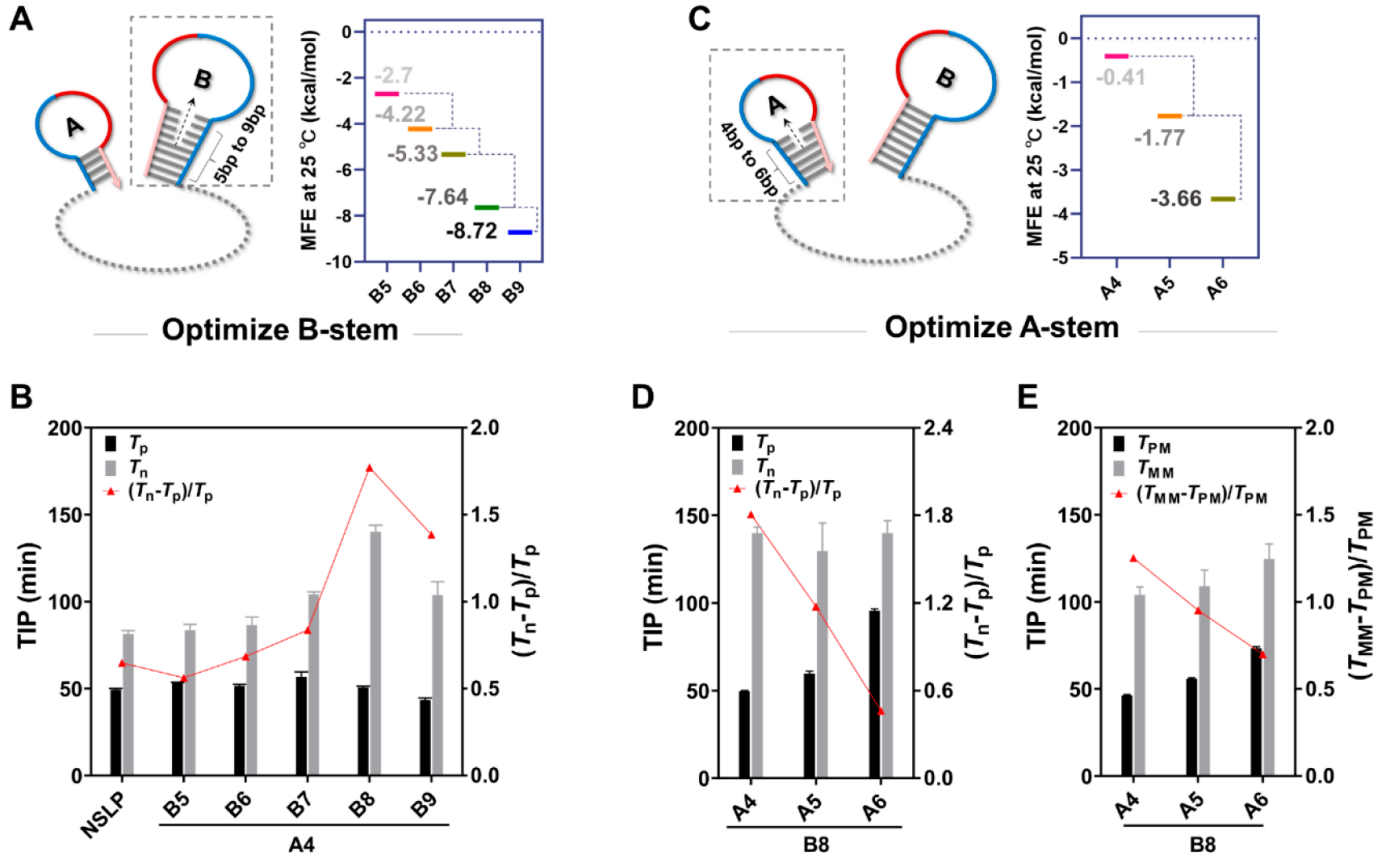
Optimization of the stem-loop structures for the DSLP. (A) Schematic of the B-stem optimization process (left) and the MFE of different B-stems (right). (B) Optimization of the B-stem length while maintaining the A-stem at 4 bp. The TIP of the amplification curves of different structural padlock probes was recorded with and without the target, *C*_miRNA_ = 500 nM, mean ± SD, *n* = 3. (C) Schematic of the A-stem optimization process (left) and the MFE of different A-stems (right). (D) Optimization of the A-stem length while maintaining the B-stem at an optimal 8 bp. (E) Effect of A-stem extension on specificity. *C*_miRNA_ = 500 nM, mean ± SD, *n* = 3.

Under optimal reaction conditions, we further tested the influence of the secondary structure of the stem-loop on the detection performance. First, we kept the stem of SL-A at 4 bp and investigated the effect of different stem lengths of SL-B. We only changed one base of SL-B adjacent to the stem every time to preserve the overall conformation of the DSLP as much as possible, making it complementary to the other base adjacent to the stem and thus increasing the length of the stem while decreasing the length of the loop. The simulated minimum free energy (MFE) gradually decreased during the above process, which meant that the stability of SL-B was effectively improved (Figure 2A). As the stem length of SL-B increased from 5 to 9 bp, (*T*_n_ – *T*_p_)/*T*_p_ first gradually increased from 56.2% to 177.1%, peaked at 8 bp, and then decreased (Figure 2B). The change in the stem length affected (*T*_n_ – *T*_p_)/*T*_p_ mainly by delaying the TIP of negative signals; that is, negative signals are more significantly suppressed with the increasing stem length of SL-B, consistent with our hypothesis that the blocked terminal stem-loop can inhibit nonspecific cyclization. Thereafter, the stem of SL-B was kept at an optimal 8 bp and the stem of SL-A increased from 4 to 6 bp in the same way (Figure 2C). We noticed that the TIP of negative signals had no obvious change, whereas positive signals delayed from 49.7 to 95.7 min, resulting in a visibly decreased (*T*_n_ – *T*_p_)/*T*_p_ value (Figure 2D). We further observed the trend of (*T*_MM_ – *T*_PM_)/*T*_PM_ because of the concern that the 4-bp A-stem was not stable enough to facilitate specificity, but the same trend as that above was obtained (Figure 2E). This phenomenon may have been due to the short toehold length initially reserved (5 bp) for SL-A. The increased stem length in SL-A further reduced the toehold/stem length ratio, which dramatically inhibited the 3′ terminal toehold-mediated strand displacement and resulted in the significant suppression of the positive signal. After a comprehensive analysis, we believe that DSLP_A4B8_ (the stems of SL-A and SL-B were 4 and 8 bp, respectively) is the optimal DSLP (referred to as A4B8 in the following context).

### Insight into the Mechanism of the DSLP Probe for Enhancing Detection Performance

Our optimal DSLP probe contained two target recognition regions, SL-A and SL-B, which recognize 9 bases at the 3′ terminal and 14 bases at the 5′ terminal of the target, respectively. We simulated the binding states of the linear (NSLP) and the stem-loop (DSLP) structures of the target recognition regions to mismatched targets using NUPAK and analyzed the effect of the stem-loop using MFE change and equilibrium concentration (EC) to observe the effect of the terminal stem-loop structure on target recognition specificity.^27^ SL-A and SL-B were first bound individually to the mismatched target. A smaller energy difference (ΔΔ*G*, 0.31 kcal/mol for SL-A, 6.25 kcal/mol for SL-B) and a lower relative EC (decreased 27.22% (T)/13.72% (M1)/1.85% (M2) for SL-A, 30.4% (T)/84.76% (M1)/73% (M2) for SL-B) were observed for the DSLP before and after binding to the mismatched targets when compared with those of the NSLP (Figures S10 and S11). The final energy of binding to the same target was almost the same with or without the stem-loop structure, and the energy difference was mainly due to the initial energy state of the target recognition sequence. A smaller energy difference (Δ*G*_2_) before and after combining targets led to a lower mismatch tolerance and a better recognition of mismatched targets. The SL-A and SL-B regions of the different structural probes were then simultaneously bound to the mismatched targets (Figure S12). Then, the relative EC was analyzed, which showed that DSLP was comparable with SSLP-A when the mismatched site was at the 3′ terminal (34.4% for SSLP-A, 36.3% for DSLP), comparable with SSLP-B when the mismatched site was at the 5′ terminal (79.4% for SSLP-B, 81.5% for DSLP), and superior to all other structures when the mismatched sites were distributed at both terminals (47.5% for NSLP, 26.9% for SSLP-A, 18.8% for SSLP-A, while DSLP is only 9.3%). The above results indicate that DSLP had the optimal target recognition ability, consistent with our initial theoretical hypothesis.

We performed DNA–protein docking and molecular dynamics (MD) simulation studies to better illustrate the influence of stem-loops on the padlock on the efficiency of ligation for the PBCV-1 DNA ligase. These simulations were based on the structure of PBCV-1 DNA ligase-adenylate bound to a 5′-phosphorylated nick (PDB: 2Q2T). This structure contained duplex DNA with a 3′-OH-5′-PO_4_ nick in the active site, where a short surface loop emanated from the oligonucleotide-binding domain (OB domain) inserted into the DNA major groove flanking the nick.^28^ Here, we removed the duplex DNA with a 3′-OH-5′-PO_4_ nick from 2Q2T and obtained the crystal structure of the PBCV-1 DNA ligase for the docking and MD with padlock probes. We chose four types of padlock probes which were exactly the same in the initial conformation and sequences to those we used in the previous experiment, and we used the HDOCK server to dock with PBCV-1 DNA ligase.^29^ The receptor binding site residues were specified according to previous reports,^30−32^ and the highest scoring representative conformation of each padlock probe was selected for the MD simulation with the AMBER14sb force field. For ∼200 ns of MD simulation, all complexes could be stabilized within 50 ns at around 0.2–0.4 nm, which indicated that all types of padlock probes could bind to the ligase and form stable conformations. For NSLP, however, stabilization of the root-mean-square deviation was delayed for ∼25 ns compared with those of other padlock probes with stem-loops. This phenomenon illustrated that the existence of stem-loops may restrain the conformational changes of a protein and influence its reactivity. The restraints aroused by the stem-loops could also be confirmed in the excessive fluctuations in the root-mean-square fluctuation. Further simulation on the hydrogen bond number revealed the steady growth of hydrogen bonds for the NSLP, and the solvent-accessible surface area indicated that no separation of the native contact was observed for all padlock probes (Figure S13). These results proved the rigidity of the active pocket and high reactivity of PBCV-1 DNA ligase when interacting with NSLP rather than DSLP.

**Figure 3:**
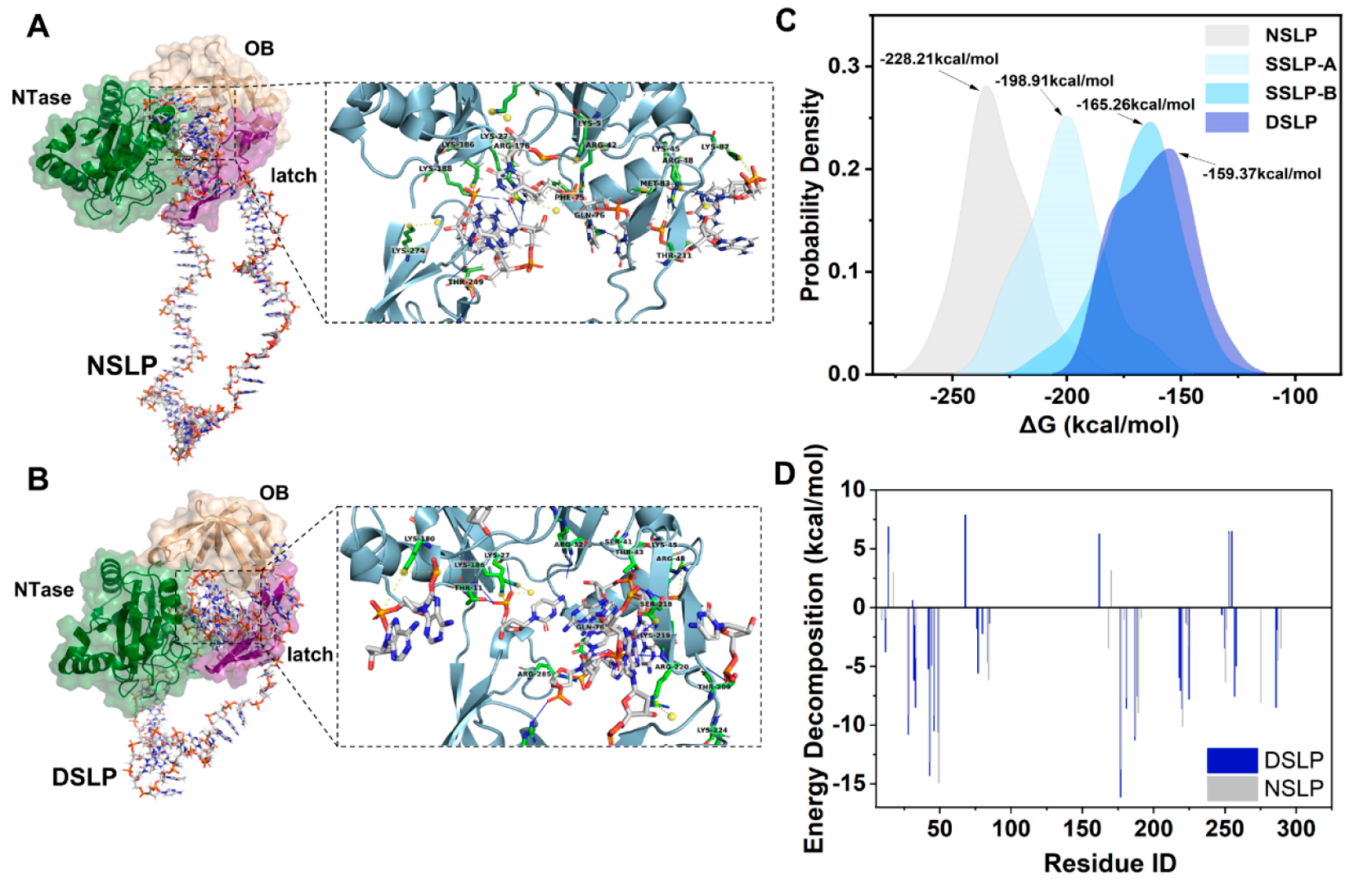
Insight into the mechanism using MD simulation. (A) Ribbon diagram of the NSLP-bound structure of the PBCV-1 DNA ligase. Green, NTase domain; beige, OB domain; magenta, latch module. The enlarged image is the stereo view of the active site of the DNA–ligase complex. (B) Ribbon diagram of the DSLP-bound structure of the PBCV-1 DNA ligase. (C) Comparison of the free binding energy distribution between the NSLP, SSLP-A, SSLP-B, and DSLP to the PBCV-1 DNA ligase using the gmx_MMGBSA method. (D) Energy decomposition for NSLP and DSLP bound to the PBCV-1 DNA ligase.

We further calculated the binding free energy between the DNA–protein complexes. The representative binding modes indicated a better interaction between ligase and NSLP (Figure 3A and 3B), and we specified the difference between the binding free energy of the solvated padlock in the bound and unbound states and compared the difference of the free energy for the solvated conformations of the same padlock probe (Table 1). The negative value of the TOTAL free energy indicated a good capture between ligase and all padlock probes. Comparing the four padlock probes, we found that the ligase–NSLP complex had the lowest free energy, −228.21 kcal/mol. With the increase in the stem-loops, the TOTAL free energy also increased gradually to −198.91 kcal/mol for SSLP-A, −165.26 kcal/mol for SSLP-B, and −159.37 kcal/mol for DSLP. The energy distribution clearly illustrated that the involvement of stem-loops for the padlock could significantly cause turbulences of the van der Waals energy and electrostatic energy, which may result in less ligation of the 3′-OH-5′-PO_4_ nick of the padlock in the absence of target (Figure 3C). A detailed comparison between DSLP and NSLP was investigated through energy decomposition, which was used to analyze the energy contribution of each residue (Figure 3D). The energy of residues G30, E67, and E160 from the NTase domain increased dramatically when interacting with DSLP, and these residues were found to form the catalytic pocket of the ligase. We believe that these results explained the relatively weak interaction between the ligase and DSLP, which gave us the opportunity to minimize negative signals in the reaction and achieve a lower limit of detection (LOD).

**Table 1:** Free Energy Analysis for the PBCV-1 DNA Ligase-Padlock DNA Complex

Ligand	Δ*G*^VDWAALS^	Δ*G*^EEL^	Δ*G*^EGB^	Δ*G*^ESURF^	Δ*G*^GGAS^	Δ*G*^GSOLV^	Δ*G*^TOTAL,^^a^
NSLP	–200.64^b^	–6923	6923.63	–28.2	–7123.64	6895.43	–228.21
SSLP-A	–173.63	–6901.63	6901.81	–25.45	–7075.26	6876.35	–198.91
SSLP-B	–183.12	–4485.8	4529.05	–25.4	–4668.92	4503.65	–165.26
DSLP	–158.76	–6342.53	6362.99	–21.06	–6501.3	6341.93	–159.37

a VDWAALS: van der Waals energy; EEL: electrostatic energy; EGB: polar solvation energy; ESURF: nonpolar solvation energy; GGAS: total gas phase free energy; GSOLV: total solvation free energy; TOTAL: GSOLV + GGAS.

b Unit: kcal/mol.

### Enhanced Sensitivity and Specificity of the DSLP Probe

**Figure 4:**
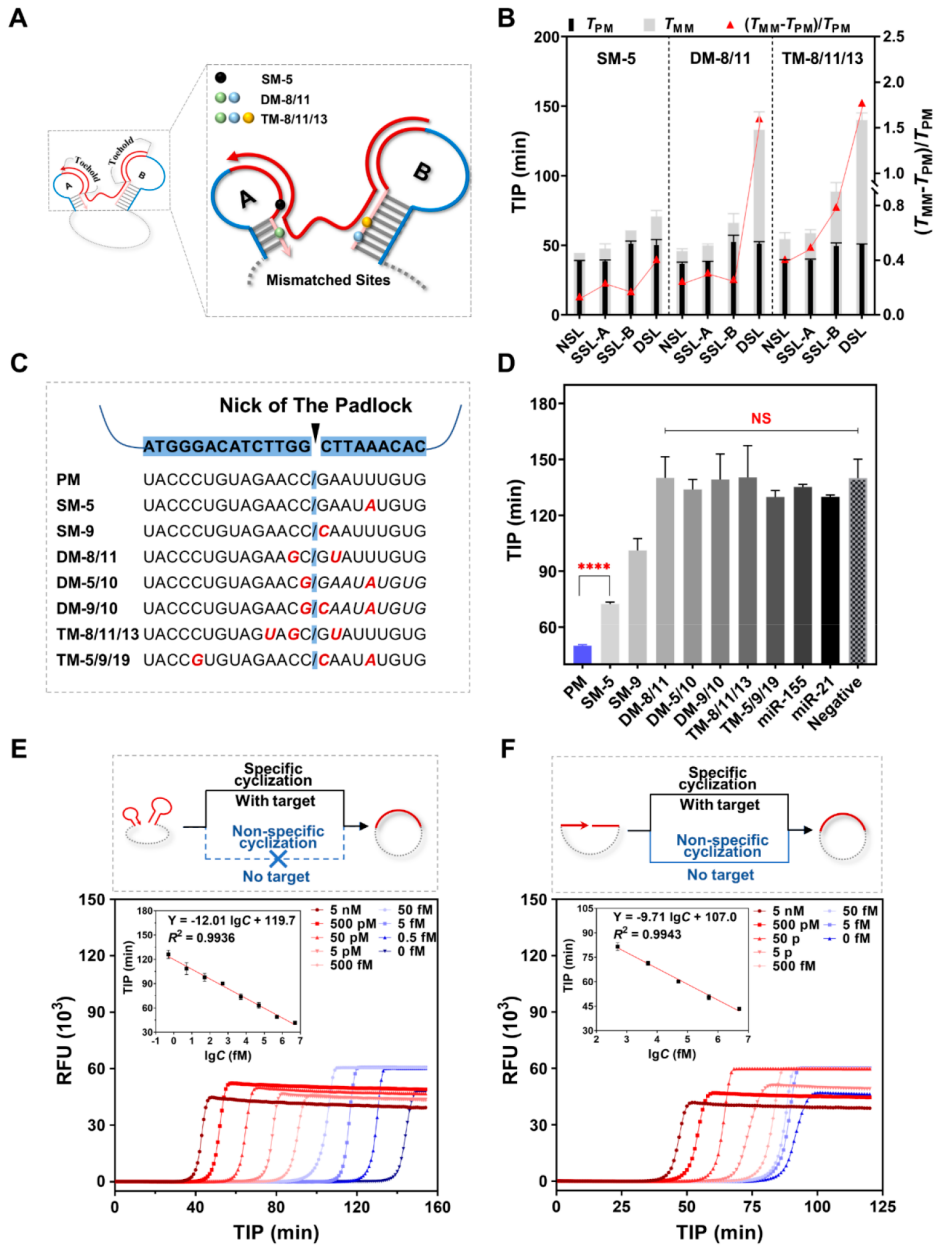
Performance comparison of the DSLP with other padlock probes. (A) Schematic of mismatched sites. SM: single-base mismatched; DM: double base mismatched; TM: triple-base mismatched. The subsequent numbers represent the positions of the mismatched sites counted from the 3′ terminal of the target. (B) Comparison of the mismatch recognition ability of different padlock probe structures. Three mismatched targets in (A) were detected using four kinds of padlock probes, *C*_miRNA_ = 500 nM, mean ± SD, *n* = 3. (C) Positions of the mismatched sites in different targets. (D) All the mismatched targets in (C) and other two miRNAs were detected using DSLP, *C*_miRNA_ = 500 nM, mean ± SD, *n* = 3. Their differences with positive (PM) and negative (without target) signals were compared separately using a two-tailed *t* test and one-way analysis of variance (ANOVA). **** denotes statistical significance (*P* < 0.0001), and ns denotes no statistical significance (*p* > 0.05). (E and F) Amplification profiles of the DSLP (E) and the NSLP (F) for different concentration targets and linear relationship between TIP and the concentration. mean ± SD, *n* = 3.

We selected the best-performing DSLP (A4B8) and compared its target-analysis performance with those of NSLP, SSLP-A, and SSLP-B to better characterize the advantages of DSLP. We first compared the mismatch recognition ability for three types of mismatched targets, whose mismatched sites are shown in Figure 4A. As the number of mismatched sites increased, the (*T*_MM_ – *T*_PM_)/*T*_PM_ of all types of padlock probes increased accordingly. This was caused by the increased instability of the mismatched bases (red curves in Figure 4B). However, the NSLP always had the lowest (*T*_MM_ – *T*_PM_)/*T*_PM_ values in all circumstances, which were 0.14 for SM-5, 0.25 for DM-8/11, and 0.41 for TM-8/11/13. With the involvement of the stem-loop structure, (*T*_MM_ – *T*_PM_)/*T*_PM_ gradually increased. Meanwhile, the DSLP structure exhibited the best recognition ability for all types of mismatches and outperformed SSLP-A and SSLP-B; the (*T*_MM_ – *T*_PM_)/*T*_PM_ values were 0.41 for SM-5, 1.60 for DM-8/11, and 1.78 for TM-8/11/13. These results demonstrated that the terminal stem-loop design facilitated the identification of base mismatches and provided the best detection specificity. Notably, regardless of whether the mismatch was in SL-A (SM-5) or equally distributed between SL-A and SL-B (DM-8/11), SSLP-A had a relatively better mismatch recognition ability than that of SSLP-B (SSLP-A and SSLP-B: 0.23 and 0.17 for SM-5, 0.31 and 0.26 for DM-8/11, respectively). This could be attributed to the fact that the target-binding region of SL-A (9 nt) was shorter than that of SL-B (14 nt), where a shorter target-binding region is more sensitive to the energy change brought by a single-base mismatch. However, the (*T*_MM_ – *T*_PM_)/*T*_PM_ of SSLP-B significantly improved from 0.26 to 0.79 and outperformed SSLP-A (0.50) when one more mismatch was added in SL-B (TM-8/11/13). This indicated that the stem-loop structure was mainly sensitive to mismatches distributed in their respective sequences. Therefore, only when SL-A and SL-B both exist, can mismatches on both sides of the nick be taken into account simultaneously, thus ensuring the effective identification of mismatches at any site of the target. We believe that this is the rationale behind the DSLP design to improve specificity.

Subsequently, the specificity of the DSLP was systematically evaluated using single-, double-, and triple-base mismatched miRNAs (two or three of each, one of which had a mismatched base at the nick site, as shown in Figure 4C) as well as other types of miRNAs (miR-155, miR-21). The results showed that miRNAs with two or more mismatched bases and other types of miRNAs had comparable signals to that of the negative control, exhibiting sufficient mismatch recognition ability. For single-base mismatches, the one at the nick site (SM-9) was better distinguished than the other one (SM-5) mainly because of the strict requirement of the ligase on base pairing at the junction (Figure 4D). Still, overall, the DSLP can effectively distinguish single-base mismatches regardless of the position of the mismatched sites.

Then, the sensitivity of the DSLP was compared with that of the conventional linear NSLP. Under the same conditions, the TIPs of the DSLP and the NSLP were delayed in accordance with the decrease of the target concentration, and both obtained a satisfactory linear relationship (*R*^2^ > 0.99) between the TIP and the target concentration (lg *C*) within a certain range (Figure 4E and 4F). However, the fluorescence curve of the DSLP could still be clearly distinguished from the negative control at the level of 0.5 fM (Figure 4E), whereas the NSLP could not be effectively distinguished when the concentration was lower than 500 fM (Figure 4F). Thus, the DSLP achieved a favorable linear relationship within 7 orders of magnitude (0.5 fM to 5 nM), whereas that of the NSLP was only within 4 orders of magnitude (500 fM to 5 nM). The wider dynamic range of detection for the DSLP was attributed to the effective suppression of the negative signal, mainly due to the inhibition of nonspecific cyclization to improve the SNR.^33^ According to the linear regression equation, the LOD was calculated using a 3-fold standard deviation (SD) of the negative control samples. The LOD for the DSLP was 133.9 aM compared to 243.9 fM for the NSLP, a more than 1000-fold increase in detection sensitivity, which was in line with our expectations. The DSLP strategy was also compared with other recent studies on biosensing for the detection of miRNAs (Table S7), and this method possessed a superior overall performance.

### The Versatility of the DSLP Design

**Figure 5:**
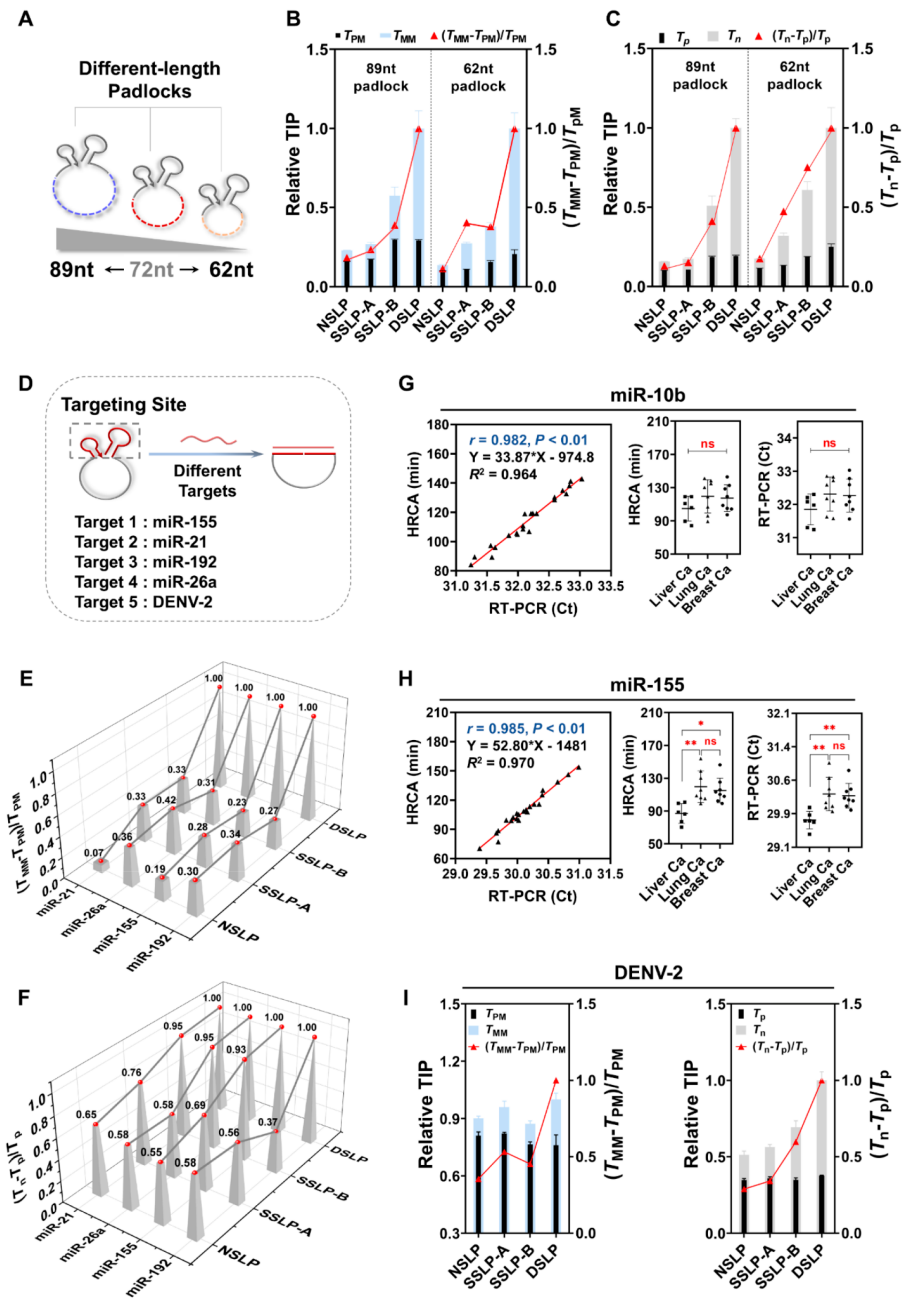
Universal validation of the DSLP probe design. (A) Schematic of different-length padlocks. (B) Comparison of the mismatch recognition abilities for different structural padlock probes with different lengths. (C) SNR comparison for different structural padlock probes with different lengths. (D) Schematic of the universal DSLP by changing only the target recognition regions (targeting site). (E) Comparison of the mismatch recognition abilities for different structural padlock probes targeting four different miRNAs. (F) SNR comparison for different structural padlock probes targeting four different miRNAs. (G and H) Detection of circulating miR-10b (G) and miR-155 (H) in plasma samples of different cancer types, indicating the concordance of the two methods. miRNA concentrations were compared between different tumors using one-way ANOVA and Tukey’s multiple comparison test, ns denotes no statistical significance (*p* > 0.05), * denotes statistical significance (*P* < 0.05), and ** denotes statistical significance (*P* < 0.01). (I) Comparison of specificity and sensitivity for different structural padlock probes targeting DENV-2.

To assess the versatility of the DSLP design, we first manipulated the length of the DSLP to be longer (89 nt) or shorter (62 nt) by extending or reducing the central portion of the padlock, which retained the same target recognition regions (Figure 5A). We optimized the length of stem-B for both padlocks and found that the optimal SNR was achieved when it featured an 8bp stem (Figure S14). Subsequently, we designed four distinct structures for both padlocks, and we performed a comparative analysis for their specificity and sensitivity. Our results indicated that the DSLP structure consistently demonstrated superior specificity and sensitivity compared to the other three structures (Figure 5B and 5C), thus underscoring the broad applicability of the DSLP strategy. We further designed five additional groups of padlock probes targeting miR-155, miR-21, miR-192, miR-25a, and DENV-2. As shown in Figure 5D, these probes were used to confirm that the DSLP design can be applied to various situations. On the basis of previous experiments, we mainly optimized the length of the B-stem from 5 to 9 bp while keeping the A-stem at a minimum length (4 or 5 bp). The results of four probes targeting miRNAs showed the same trend and achieved maximum (*T*_n_ – *T*_p_)/*T*_p_ when the B-stem length was increased to be stable enough (approximately 8 bp) (Figure S15). This phenomenon was consistent with previous results, implying the importance of robust closure at the terminal of SL-B for suppressing negative signals.

Optimal DSLP probes were selected and compared to three other types of padlock probes (SSLP-A, SSLP-B, and NSLP). First, the detection specificity was examined using single-base mismatched targets. As expected, the (*T*_MM_ – *T*_PM_)/*T*_PM_ values of all DSLP probes were more than twice those of the other types of padlock probes (Figure 5E). The significantly higher (*T*_MM_ – *T*_PM_)/*T*_PM_ values in all groups demonstrated the versatility of the DSLP structure in improving specificity. Then, (*T*_n_ – *T*_p_)/*T*_p_ was analyzed to evaluate the detection sensitivity (Figure 5F). Notably, except for the SSLP-B of miRNA-192, (*T*_n_ – *T*_p_)/*T*_p_ increased with the total number of complementary bases of the stem-loops. This further demonstrated the importance of the stability of stem-loops in effectively blocking terminals. The (*T*_n_ – *T*_p_)/*T*_p_ values of the DSLP probes all remained at the highest level, and the optimal SNR meant the highest detection sensitivity. The TIP of the amplification curves (Figures S16–S19) showed that the difference between the positive signals (*T*_p_ and *T*_PM_) in each group was not significant compared with those of the negative signals (*T*_n_) or mismatched target signals (*T*_MM_). This suggests that the stem-loop structure improved the detection sensitivity and specificity mainly by suppressing negative or mismatched target signals. This is consistent with our previous studies of the performance of padlock probes targeting miR-10b.

Next, we collected a total of 22 plasma samples from three different tumor patients (liver cancer, lung cancer, and breast cancer) to verify the consistency of the HRCA signal of DSLP with the golden standard—the reverse transcription polymerase chain reaction (RT-PCR). miR-10b and miR-155 were detected for the proof of concept. The levels of the two miRNAs tested using DSLP-based HRCA were in good consistency with those tested using RT-PCR (*R*^2^ values (linear regression) of 0.964 and 0.970, r values (Pearson correlation) of 0.982 and 0.985, respectively), indicating the potential clinical practicability of the method. Thereafter, we analyzed the variability of the results among samples from different tumors. Similar results were obtained from the two methods; that is, miR-10b concentrations were not significantly different among the three tumors, whereas the expression of miR-155 was significantly higher in liver cancer than in the other cancer types (Figure 5G and 5H). This reveals that our method has comparable detection capability with that of RT-PCR and also shows the limitations of using a single miRNA to diagnose and differentially diagnose tumors.^34^ We also validated the versatility of our strategy for longer target detection, such as the conserved 60 nt sequence of DENV-2. The length of the B-stem was optimized (Figure S20), and a comparison of the specificity and sensitivity between different padlock structures indicated that the DSLP also exhibited superior capability (Figure 5I).

### Application in Multiplex Detection of miRNAs

Previous reports have shown that the combined detection of multiple circulating miRNAs significantly improves diagnostic efficacy.^35−37^ Motivated by the limitations of single miRNA detection, we further explored the application of DSLP-based HRCA in multiplex detection. Theoretically, multiple padlock probes with different target recognition regions can target multiple miRNAs to form specific circular chains at corresponding concentrations in a one-pot reaction. Then, distinguishing the signals of different circular chains in the same reaction system is the key to achieving multiplex detection. Different signals or the time and space difference of the same signal can be used to distinguish multiple targets.^38−40^

**Figure 6:**
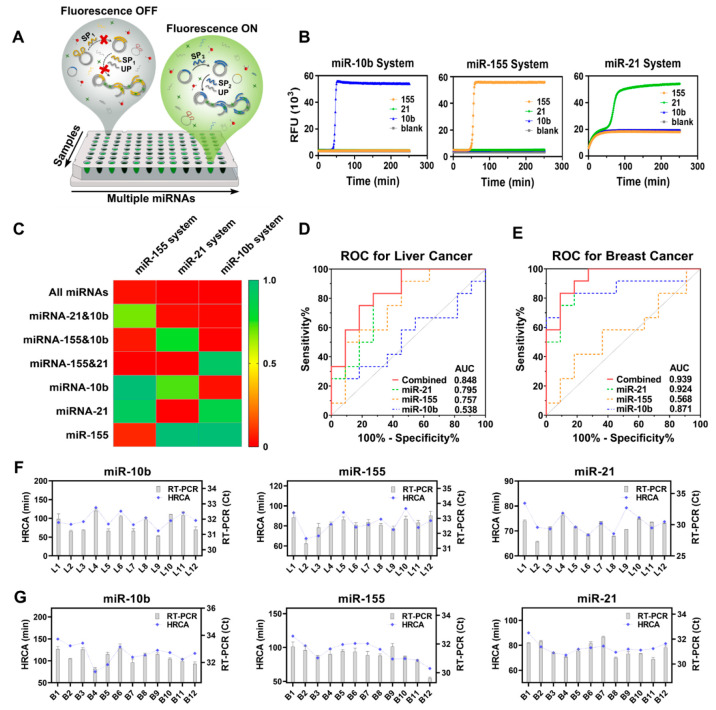
DSLP-based HRCA for multiplex detection. (A) Schematic of multiplex fluorescent signal outputs. (B) Amplification curves of different detecting systems with cyclization products of three miRNAs, *C*_miRNA_ = 500 nM. (C) Multiplex detection of synthetic samples using DSLP-based HRCA; *C*_miRNA_ = 500 nM. (D and E) ROC curves of miR-10b, miR-155, miR-21, and a combination of the three miRNAs for liver cancer (D) and breast cancer (E). The concentration of miRNAs obtained from healthy donors (*n* = 11), liver cancer patients (*n* = 12), and breast cancer patients (*n* = 12) via our multiplex detection system. (F and G) Consistency analyses of our multiplex detection system and RT-PCR. L: liver cancer; B: breast cancer; the subsequent numbers represent the sample number.

In this study, the space difference in the HRCA signal was chosen. We designed specific primers (SPs) and the universal primer (UP) targeting the target recognition regions and functional regions of the DSLP, respectively. The target recognition regions are specific sequences for each circular chain; therefore, the SPs can initiate specific amplifications by recognizing these regions. As shown in Figures 6A and S22A, on a two-dimensional (2D) array plane, each column contains one specific HRCA system (with one selected SP) corresponding to one specific circular chain. If the DSLP probes corresponding to the SP are not cyclized into rings, then the SP-initiated amplification cannot proceed, and no fluorescent signal could be detected. In contrast, the fluorescent signal could be detected in the presence of the target. Thus, when one sample is added to multiple columns simultaneously, the position and intensity of the fluorescence signal could represent the target and corresponding concentration contained in the sample.

We first investigated the impact of an uncyclized DSLP probe on the background signal of the HRCA system and confirmed that the background signal generated by an uncyclized DSLP probe at the experimental concentration can be disregarded (Figure S21). Subsequently, to verify the recognition specificity of SPs, cyclization products of three miRNAs were added to three HRCA systems and the amplification profiles of each system were recorded. Only the cyclized product corresponding to the detection system generated an effective fluorescence signal, whereas the others had faint signals comparable to that of the blank control (Figure 6B). This demonstrated that the SPs can effectively identify respective circular chains. Next, different combination samples of three synthetic miRNAs were prepared to perform multiplex detection using the proposed scheme (Figures 6C and S22B). The heatmap showed that only the contained miRNAs that were consistent with the HRCA system could generate positive fluorescence signals. The fluorescence signal of the mixed target was comparable to that of the single target, and that of the interfering target was comparable to that in the absence of the target. The outstanding distinguishing ability of different targets and their combinations proved that DSLP-based HRCA was capable of multiplex detection.

We tested the multiplex detection system for clinical practice as a proof of concept. Plasma samples from healthy individuals (*n* = 11), liver cancer patients (*n* = 12), and breast cancer patients (*n* = 12) were involved, and miR-10b/155/21 were tested using both our multiplex scheme and RT-PCR. Fluorescence signals of the three different miRNAs in one sample were obtained simultaneously using our multiplex method, and the receiver operating characteristic (ROC) curve of miR-10b, miR-155, miR-21, and a combination of the three miRNAs for liver cancer and breast cancer was analyzed (Figure 6D and 6E). The area under the curve (AUC) of a single miRNA was 0.538 (miR-10b), 0.757 (miR-155), and 0.795 (miR-21) for liver cancer and 0.871 (miR-10b), 0.562 (miR-155), and 0.924 (miR-21) for breast cancer. When the three miRNAs were combined by binary logistic regression, the AUC increased to 0.848 for liver cancer and 0.939 for breast cancer, effectively improving the diagnostic performance and demonstrating the diagnostic potential of multiplex miRNAs’ detection. Meanwhile, the comparison between our multiplex detection system and RT-PCR showed a good correlation (Figure 6F and 6G),^41^ further demonstrating the reliability of the multiplex detection system. Our multiplex detection system can be expanded in 2D space to detect more targets simultaneously, providing a technical platform to effectively monitor circulating miRNA panels in various diseases. In this study, we only verified the feasibility of our method for multiplex detection with 2D arrays; nevertheless, our method can be combined with other multiplex detection methods, such as suspension microarrays, to build a more powerful and practical multitarget detection platform, contributing to the development of molecular diagnostics.

## Conclusions

In this work, we explored a simple structural reconstruction method to improve both the specificity and sensitivity of padlock-assisted RCA, which was developed into a multiplexed detection technology platform of circulating miRNA panels and showed excellent diagnostic efficacy in the diagnosis of breast and liver cancers. We reconstructed only the two terminals of the conventional NSLP and preserved the integrity of the functional region, which facilitated the integration of our reconstruction strategy with the needs of other experimental designs. By comparison of different structures of padlock probes, the feasibility of the DSLP design for improving the detection performance was verified experimentally and theoretically. The simulation results showed that the stem-loop structure with a lower free energy reduced the energy difference in the target recognition process, leading to a lower mismatch tolerance and thus improving the detection specificity. The delayed negative signal of the DSLP in HRCA and a weaker binding of the DSLP to the ligase in MD simulations were observed. These phenomena proved that the presence of stem-loops effectively inhibited nonspecific cyclization in the absence of the target by steric hindrance, which effectively suppressed negative signals and improved the detection sensitivity. Taking miR-10b as a model target, the DSLP obtained a prior single-base discrimination ability compared with other structures and reached the aM-level detection limit, which was over 1000-fold higher than that of the conventional NSLP. Subsequently, the versatility of the design was validated using longer and shorter padlocks targeting the same target, as well as five additional targets. A similar signal change trend and an improvement in both sensitivity and specificity were observed in all cases, which indicated the universality of our design strategy. Finally, we established a multiplex detection system for DSLP-based HRCA and demonstrated its clinical practicability by testing plasma samples from multiple cancer patients. The findings and knowledge gained from this work can give us a more comprehensive understanding of the impact of nucleic acid structure on detection and provide another perspective for developing highly specific and sensitive detection systems. We believe that a thorough investigation of the mechanisms behind the interaction between nucleic acids or between nucleic acids and enzymes can enhance the efficacy of practical detection strategies and advance clinical diagnostic research.

## Methods/Experimental Section

### Design and Analysis of Padlock Probes

The design and analysis of padlock probes was performed using the Nucleic Acid Package (NUPACK, http://www.nupack.org) at 25 °C (experimental reaction temperature).^42,43^ A conventional linear padlock probe consists of two target-recognition regions (at both terminals), one functional region (in the middle), and two spacers between them. The two target-recognition regions were complementary to the target miRNA and can be changed to recognize any target of interest. The functional region was a freely editable sequence, which can be designed into different structures according to experimental needs. In this work, the functional region was designed as a C-rich sequence that could be transcribed to form a G-quadruplex. Only part of the “spacer” of the conventional padlock probe was edited to be the “accessory”, which was used to assist the “target recognition” to form the terminal stem-loop secondary structure, so that reconstructed padlock probes retained the same functional region and target recognition regions as the conventional padlock. We finally successively built four kinds of padlock probes with different secondary structures. According to whether there was a stem-loop at the 3′ or 5′ terminal, they were named: non-stem-loop padlock (NSLP, without stem-loop), single-terminal stem-loop padlock A (SSLP-A, with one stem-loop at the 3′ terminal), single-terminal stem-loop padlock B (SSLP-B, with one stem-loop at the 5′ terminal), and dual-terminal stem-loop padlock (DSLP, with two stem-loops at both terminals). The full length of a padlock was 72 nt, and the sequences forming stem-loops A and B (SL-A and SL-B) were fixed at 17 nt and 26 nt, respectively. SL-A and SL-B were the target recognition regions, the stem length of which could be altered to suit experimental needs.

To simulate the binding state of a target to SL-A and SL-B, the sequences of SL-A (17 nt) and SL-B (26 nt) binding to the target respectively and simultaneously at 25 °C were analyzed using UNPACK. The equilibrium concentration (EC) and minimum free energy (MFE) were collected.^44^

### Cyclization of Padlock Probes

Cyclization of padlock probes was a recognition process of targets. Padlock probes with different structures were ligated into circular probes in the presence of the target miRNA. To achieve the best performance, the concentrations of the padlock probe and buffer and also reaction time were optimized. Unless otherwise indicated, the cyclization was typically performed by mixing a certain concentration of target miRNA and 25 nM padlock probe in a 20 μL reaction mixture, containing 1 μL (25 U/μL) PBCV-1 DNA ligase, 1 μL (40 U/μL) Recombinant RNase Inhibitor, and 0.05X PBCV-1 DNA ligase Reaction Buffer (2.5 mM Tris-HCl, 0.5 mM MgCl_2_, 0.05 mM ATP, 0.5 mM DTT, pH 7.5, at 25 °C). For multiplex detection, several types of padlock probes targeting different miRNAs were all added to the cyclization system simultaneously (25 nM each), and other conditions remained unchanged. The reaction mixture was made up on ice, transferred rapidly into a dry bath incubator, then incubated at 25 °C for 15 min, and inactivated at 65 °C for 20 min. To evaluate the effect of the cyclization, the noncyclized padlock probes were removed using 2 μL (20 U/μL) of Exonuclease I and 2 μL (100 U/μL) of Exonuclease III (total 20 μL) and then electrophoresed on 10% denatured polyacrylamide gel (110 V, 65 min).

### Real-Time HRCA

After cyclization, the resulting solution (5 μL) was mixed with 0.8 μL (10 μM) of specific primer (SP, complementary to the target recognition region to trigger HRCA) and 0.8 μL (10 μM) of universal primer (UP, the sequence same as functional region, cooperated with SP to form hyper branches), 0.6 μL (each 10 mM) of dNTP mixture, 1 μL (8 U/μL) of Bst 2.0 DNA Polymerase, 2 μL of 20X AugeGreen, 2 μL of 10X Isothermal Amplification Buffer (20 mM Tris-HCl, 10 mM (NH_4_)_2_SO_4_, 50 mM KCl, 2 mM MgSO_4_, 0.1% Tween 20, pH 8.8, at 25 °C), and 7.8 μL of DNase/RNase-free deionized water. This process was done in a 200 μL PCR tube on ice and then were transferred to a CFX96 Real-Time System at 55 °C, and fluorescence was monitored at 1 min intervals.

### Molecular Dynamics Simulation

HDock was used to dock the protein (PDB: 2Q2T) and the nucleic acids.^45^ The four types of padlock probes used in the simulation were identical with those designed for the experiment. To ensure the consistency of our simulations, we employed Tiamat DNA Editor 2 (https://www.public.asu.edu/~hyan6/Resources.html) and ChimeraX (Version 1.4rc202205111743, https://www.rbvi.ucsf.edu/chimerax) to generate padlocks with the same initial conformations and sequences as NSLP, SSLP-A, SSLP-B, and DSLP.^46^ The docking results were taken as the initial conformation of the kinetic simulation. Gromacs 2019.6 was selected as the kinetic simulation software, and the amber14sb force field was applied.^47^ The TIP3P water model was used to establish a water box for the complex system, and a sodium ion was added to equilibrate the system. The elastic simulation was treated by the Verlet and CG algorithm, the electrostatic interaction was treated with the PME (Particle-mesh Ewald) method, and the steepest descent method was used for the energy minimization within the maximum steps of 50000. The Kulun force and the van der Waals force cutoff distance were both 1.4 nm. Finally, the system was equilibrated by the NVT and NPT system and then applied for the molecular dynamic simulation within 200 ns at room temperature and atmospheric pressure. During the simulation, the LINCS algorithm was used to constrain the related hydrogen bonds, and the integration time step was 2 fs. Root mean square deviation (RMSD) and root-mean-square fluctuation (RMSF) were used to describe the local allosteric effect during the simulation (the fluctuation cutoff was set at 0.2). The solvent accessible surface area (SASA) was used to describe the size of the solvent-accessible surface area of the complex during the simulation. Hydrogen bond number (HBNUM) was used to describe the number of formed hydrogen bonds between the protein and the nucleic acid during the simulation.

### Binding Energy Calculation

Molecular trajectories were used for the binding energy calculation:
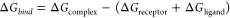
1
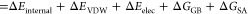
2where Δ*E*_internal_ is the internal energy, Δ*E*_VDW_ is the van der Waals potential energy, Δ*E*_elec_ is the electrostatic potential energy, Δ*G*_GB_ is the polar solvation free energy, and Δ*G*_SA_ is the nonpolar solvation free energy. Here Δ*G*_GB_ was calculated by the generalized Born solvent model which was developed by Nguyen et al.^48^ Δ*G*_SA_ was calculated by the product of surface tension and solvent accessibility surface area, which was ΔΔ*G*_SA_ = 0.0072 × Δ*S*ASA.^49^ Entropy change was ignored in this study due to the high consumption of computational resources and low precision. This algorithm is implemented by gmx _MMPBSA.^50^

### Polyacrylamide Gel Electrophoresis Analysis

To accurately quantify the cyclization efficiency of padlock probes with different structures, we analyzed the band intensity on the PAGE gel using Image Lab software (Version 5.2, build 14, Bio-Rad). Briefly, images of the lanes and bands on the gel were acquired by using automated scanning first. Then all the bands were marked and the reference band designated. After that, relative quantification of each band was recorded in the analysis table, and the average band intensity was calculated.

### RCA and G-Quadruplex Catalytic Reaction

Following cyclization, RCA reactions (20 μL) were initiated by 0.6 μL (each 10 mM) of dNTP Mixture, 1 μL (10U/μL) of phi29 DNA polymerase (M0269L, NEB), 0.1 μL (20 ug/μL) of BSA (B9200S, NEB), 0.2 μL (0.1 U/μL) of IPP (M0361S, NEB), and 1X phi29 DNA Polymerase Reaction Buffer (50 mM Tris-HCl, 10 mM MgCl_2_, 10 mM (NH_4_)_2_SO_4_, 4 mM DTT, pH 7.5, at 25 °C) at 37 °C for 90 min and inactivated at 65 °C for 10 min. Then, RCA products (20 μL) were incubated with 10 μL of KCl (500 mM) and 20 μL of 1X TE at 95 °C for 10 min and then at 37 °C for 30 min. After that, 2 μL of hemin (1 mM) was added and mixed well and then the reaction mixture was continued to incubate at 37 °C for 30 min. At the same time, H_2_O_2_ and TMB were mixed in a ratio of 1:1 to form a chromogenic substrate. 50 μL of the chromogenic substrate was added into the above incubated solution to observe its color change using the naked eye. Within 10 to 30 min, the optical densities at 350∼700 nm were recorded at the end of incubation.

### Clinical Plasma Samples

In total, we collected 57 EDTA-anticoagulated fresh whole blood samples, including those from 11 healthy individuals, 18 liver cancer patients, 8 lung cancer patients, and 20 breast cancer patients, from Southwest Hospital. All blood samples were centrifuged at 1600*g* for 10 min as soon as possible, and then plasma was separated into fresh RNase-free tubes and frozen at −80 °C for later use. This study was approved by the Ethics Committee of First Affiliated Hospital, Army Medical University, and performed in accordance with the Declaration of Helsinki and the International Ethical Guidelines for Biomedical Research Involving Human Subjects.

### Total RNA Extraction

250 μL of serum was pipetted into a 2 mL tube and 750 μL of RNAiso Blood was added, mix well by pipetting up and down, and then left to stand for 5 min at room temperature. Subsequently, 200 μL of chloroform was added to the solution, and the resulting mixture was shaken vigorously for 15 s, placed at rest for 5 min at room temperature, and centrifuged at 12000*g* for 15 min at 4 °C. The upper aqueous phase was aspirated carefully into a new tube, an equal volume of cold isopropanol was added to the aqueous solution, and the mixture was mixed thoroughly upside down to precipitate the RNA, and then the sample was put at −20 °C and left to stand for 20 min and centrifuged at 12000*g* for 10 min at 4 °C. The supernatant was removed, the RNA pellet was washed with an equal volume of 75% ethanol once, the resulting mixture was centrifuged at 7500*g* for another 10 min at 4 °C, and then the ethanol was discarded after the centrifugation. The RNA pellet was air-dried for 20 min and dissolved in DNase/RNase-free water. The concentration was determined by a NanoDrop One microvolume UV–vis spectrophotometer (Thermo Fisher).

### Reverse Transcription PCR (RT-PCR)

The stem-loop RT-PCR method was used as a standard method to analyze the concentration of miRNAs. A miRNA First Strand cDNA Synthesis Kit was used to prepare the complementary DNA (cDNA). For a 20 μL reverse transcription reaction system, 1.5 μL of miRNA L-RT enzyme mix, 10 μL of 2X miRNA L-RT solution mix, 1 μL of 10 μM primer, and 3 μL of total RNA solution were mixed in a 200 μL PCR tube on ice, then incubated at 16 °C for 30 min and 37 °C for 30 min, deactivated at 85 °C for 5 min, and stored at 4 °C. For a 20 μL PCR system, 2 μL of DNF buffer, 10 μL of 2X SG fast qPCR master mix, 0.4 μL of 10 μM forward and reverse primers, 6 μL of cDNA product, and 1.2 μL of H_2_O were mixed in a 200 μL PCR tube on ice and then transferred to a CFX96 Real-Time System, and PCR was run according to the manufacturer’s manual: (1) activation at 95 °C for 3 min; (2) denaturation at 95 °C for 3 s, annealing at 60 °C for 30 s, 40 cycles; (3) measure melting curve from 65 to 95 °C with a 0.5 °C increment.

## Supporting Information Available

Chemicals and apparatuses, oligonucleotide sequences, comparison of recent studies on the detection of miRNAs, and supplementary figures showing denatured PAGE for the process of DSLP reaction; feasibility verification and condition exploration for G-quadruplex catalytic property; validation for the catalytic efficacy of the functional region of DSLP as the G-quadruplex and as an HRCA primer site; comparison of cyclization reactions and HRCA reactions for the NSLP and the DSLP; effect of different ligation buffer concentrations, different concentrations of padlock probe, different cyclization temperatures, and different reaction times; NUPACK simulation for the binding state of the SL-A region, the SL-B region, or the SL-A and SL-B regions simultaneously to mismatched targets; MD simulations of padlocks with four structures; optimization of the B-stem length for DSLP probes; comparison of mismatch recognition and SNRs for different structural padlocks for miRNA detection; amplification curves of miR-10b HRCA systems; and feasibility verification of multiplex detection based on DSP probes (PDF)

## Abbreviations

RCA: rolling circle amplification
HRCA: hyperbranched RCA
SNR: signal-to-noise ratio
FRET: fluorescence-resonance-energy-transfer
NSLP: non-stem-loop padlock
DSLP: dual-terminal stem-loop padlock
SSLP-A: single-terminal stem-loop padlock A
SSLP-B: single-terminal stem-loop padlock B
PAGE: polyacrylamide gel electrophoresis
TIP: time of inflection point
MFE: minimum free energy
EC: equilibrium concentration
MD: molecular dynamics
RMSD: root-mean-square deviation
RMSF: root-mean-square fluctuation
HBNUM: hydrogen bond number
SASA: solvent-accessible surface area
LOD: lower limit of detection
SD: standard deviation
RT-PCR: reverse transcription polymerase chain reaction
SP: specific primer
UP: universal primer
ROC: receiver operating characteristic
AUC: area under the curve
2D: two-dimensional
