# Supplementary material for: A Universal Strategy for Enhancing the Circulating miRNAs’ Detection Performance of Rolling Circle Amplification by Using a Dual-Terminal Stem-Loop Padlock
Source: ACS Nano. 2023 Dec 27;18(1):436–50. doi: 10.1021/acsnano.3c07721 (PMC10786163; doi:10.1021/acsnano.3c07721)
Supplement: Supplementary file 1 — nn3c07721_si_001.pdf [file nn3c07721_si_001.pdf]

Supporting Information

for

A Universal Strategy for Enhancing The Circulating miRNAs Detection Performance of Rolling Circle Amplification by Using Dual-Terminal Stem-Loop Padlock

Hanqing Xu [a‡], *Xianlan Wu* [a‡], *Qian Liu* [a‡], *Cheng Yang* [a], *Man Shen* [a], *Yingran Wang* [a], *Shuai Liu* [a], *Shuang Zhao* [a], *Ting Xiao* [a], *Minghui Sun* [a], *Zishan Ding* [a], *Jing Bao* [a*], *Ming Chen* [ab*], *Mingxuan Gao* [a*]

[a] Department of Clinical Laboratory Medicine, Southwest Hospital, Third Military Medical University (Army Medical University), Chongqing, 400038, P. R. China

[b] College of Pharmacy and Laboratory Medicine, Third Military Medical University (Army Medical University), 30 Gaotanyan, Shapingba District, Chongqing 400038, P. R. China.

[*]Corresponding Author: Jing Bao, Ming Chen, Mingxuan Gao. **Email:** baojing_1991@163.com, chming1971@126.com, mingxuan_gao@163.com.

[‡]*Hanqing Xu, Xianlan Wu, Qian Liu* contributed equally.

Table of Contents

Materials and Methods 3

Supplementary Tables 12

Supplementary Figures 21

Supplementary Reference 53

Materials and Methods

Chemical and instruments

All oligonucleotides were purified by high-performance liquid chromatography (HPLC) and synthesized by Sangon Biotech Co., Ltd. (Shanghai, China), shown in Tables S1~S6. The lyophilized powder of DNA and RNA was dissolved in DNase/RNase-free deionized water, diluted to 10 μM, and stored at -20 °C. All enzymes were obtained from New England Biolabs Lnc. (MA, USA). Recombinant RNase Inhibitor (2313A), dNTP Mixture (4019), and RNAiso Blood kit for total RNA extraction (9112/9113) were supplied by TaKaRa Biotechnology (Dalian, China). Potassium chloride (KCl) and hemin (in DMSO) were purchased from Solarbio Life Sciences Co., Ltd. (Beijing, China). GelRed Nucleic Acid Gel Stain and AugeGreen (20X in water) were acquired from US EverBright Lnc. (Suzhou, China). Polyacrylamide gel kit was bought from Beyotime Biotechnology Lnc. (Shanghai, China). DNase/RNase-free deionized water supplied by Tiangen Biotech Co.,Ltd. (Beijing, China). 2X TBE-Urea Sample Buffer, 5X TBE Buffer, 1X TE Buffer, TMB Chromogen Solution and Reverse transcription PCR analysis kit were purchased from Sangon Biotech Co., Ltd. (Shanghai, China). Blood from patients with cancer (lung, liver, breast cancer) was collected from Southwest Hospital of Army Medical University (Chongqing, China), and this study was performed under the supervision of the Ethics Committee of the hospital. Unless otherwise indicated, all chemicals were used as received.

Experiments requiring constant incubation temperatures, such as cyclization, digestion, and RCA, were carried out in a dry bath incubator (TIANGEN, Beijing, China). Electrophoresis was performed using the gel electrophoresis system (Bio-Rad, CA, USA). Images of PAGE were captured and analyzed on the ChemiDoc MP imaging system (Bio-Rad, CA, USA). Real-time fluorescence detection of hyperbranched RCA and RT-PCR was carried out on a CFX96 Real-Time System (Bio-Rad, CA, USA).

Design and analysis of padlock probes

The design and analysis of padlock probes was performed using the Nucleic Acid Package (NUPACK, http://www.nupack.org) at 25 °C (experimental reaction temperature).[1, 2] A conventional linear padlock probe consists of two target-recognition regions (at both terminals), one functional region (in middle), and two spacers between them. The two target-recognition regions were complementary to the target miRNA and can be changed to recognize any target of interest. The functional region was a freely editable sequence, which can be designed into different structures according to experimental needs. In this work, the functional region was designed as a C-rich sequence that could be transcribed to form a G-quadruplex. Only part of the “spacer” of the conventional padlock probe was edited to be the “accessory”, which was used to assist the “target recognition” to form the terminal stem-loop secondary structure, so that reconstructed padlock probes retained the same functional region and target recognition regions as the

conventional padlock. We finally successively built four kinds of padlock probes with different secondary structures. According to whether there was a stem-loop at the 3′ or 5′ terminal, they were named: non-stem-loop padlock (NSLP, without stem-loop), single-terminal stem-loop padlock A (SSLP-A, with one stem-loop at 3′ terminal), single-terminal stem-loop padlock B (SSLP-B, with one stem-loop at 5′ terminal) and dual-terminal stem-loop padlock (DSLP, with two stem-loops at both terminals). The full length of a padlock was 72 nt, and the sequences forming the stem-loops A and B (SL-A and SL-B) were fixed at 17 nt and 26 nt, respectively. SL-A and SL-B were the target recognition regions, the stem length of which could be altered to suit experimental needs.

To simulate the binding state of a target to SL-A and SL-B, the sequences of SL-A (17 nt) and SL-B (26 nt) bind to the target respectively and simultaneously at 25 °C was analyzed using UNPACK. The equilibrium concentration (EC) and minimum free energy (MFE) were collected[3].

Cyclization of padlock probes

Cyclization of padlock probes was a recognition process of targets. Padlock probes with different structures were ligated into circular probes in the presence of the target miRNA. To achieve the best performance, the concentration of the padlock probe and buffer, also reaction time were optimized. Unless otherwise indicated, the cyclization was typically performed by mixing a certain concentration of target miRNA and 25 nM padlock probe in a 20 μL reaction mixture, containing 1 μL (25

U/μL) PBCV-1 DNA ligase, 1 μL (40 U/μL) Recombinant RNase Inhibitor and 0.05X PBCV-1 DNA ligase Reaction Buffer (2.5 mM Tris-HCl, 0.5 mM $MgCl_2$, 0.05 mM ATP, 0.5 mM DTT, pH 7.5, at 25 °C). For multiplex detection, several types of padlock probes targeting different miRNAs were all added to the cyclization system simultaneously (25 nM each), other conditions remained unchanged. The reaction mixture was made up on ice and transferred rapidly into a dry bath incubator, then incubated at 25 °C for 15 min and inactivated at 65 °C for 20 min. To evaluate the effect of the cyclization, the non-cyclized padlock probes were removed using 2 μL (20 U/μL) Exonuclease I and 2 μL (100 U/μL) Exonuclease III (total 20 μL), and then electrophoresed on 10% denatured polyacrylamide gel (110 V, 65 min).

Real-time HRCA

After cyclization, mixed the resulting solution (5 μL) with 0.8 μL (10 μM) specific primer (SP, complementary to the target recognition region to trigger HRCA) and 0.8 μL (10 μM) universal primer (UP, the sequence same as functional region， cooperated with SP to form hyper branches), 0.6 μL (each 10 mM) dNTP Mixture, 1 μL (8 U/μL) Bst 2.0 DNA Polymerase, 2 μL 20X AugeGreen, 2 μL 10X Isothermal Amplification Buffer (20 mM Tris-HCl, 10 mM $(NH_4)_2SO_4$, 50 mM KCl, 2 mM $MgSO_4$, 0.1% Tween 20, pH 8.8, at 25 °C) and 7.8 μL DNase/RNase-free deionized water. This process was done in a 200 μL PCR tube on ice, then were transferred to a CFX96 Real-Time System at 55 °C and monitored fluorescence at 1min intervals.

Molecular dynamics simulation

HDock was used to dock the protein (PDB: 2Q2T) and the nucleic acids. [4] The four types of padlock probes used in the simulation were identical to those designed for the experiment. To ensure the consistency of our simulations, we employed Tiamat DNA Editor 2 (https://yanlab.asu.edu/Resources.html) and ChimeraX (Version 1.4rc202205111743, https://www.rbvi.ucsf.edu/chimerax) to generate padlocks with the same initial conformations and sequences as NSLP, SSLP-A, SSLP-B, and DSLP.[5] The docking results were taken as the initial conformation of the kinetic simulation. The Gromacs 2019.6 was selected as the kinetic simulation software, and the amber14sb force field was applied. [6]The TIP3P water model was used to establish a water box for the complex system, and the sodium ion was added to equilibrate the system. The elastic simulation was treated by Verlet and CG algorithm, the electrostatic interactions was treated with PME (Particle-mesh Ewald) method, steepest descent method was used for the energy minimization within the maximum steps of 50000. The Kulun force and the Van Der Waals force cutoff distance was both 1.4 nm. Finally, the system was equilibrated by NVT and NPT system, then applied for the molecular dynamic simulation within 200 ns at room temperature and atmospheric pressure. During the simulation, LINCS algorithm was used to constrain the related hydrogen bonds, the integration time step is 2 fs. Root mean square deviation (RMSD) and root mean square fluctuation (RMSF) were used to describe the local allosteric effect during the simulation (the fluctuation cutoff was set at 0.2). The Solvent accessible surface area (SASA) was

used to describe the size of the solvent-accessible surface area of the complex during the simulation. Hydrogen bond number (HBNUM) was used to describe the number of formed hydrogen bond between the protein and the nucleic acid during the simulation.

Binding energy calculation

Molecular trajectories were used for the binding energy calculation:

$$\Delta G_{bind} = \Delta G_{\mathrm{complex}} - (\Delta G_{\mathrm{receptor}} + \Delta G_{\mathrm{ligand}}) \quad (1)$$

$$= \Delta \mathrm{E}_{\mathrm{internal}} + \Delta \mathrm{E}_{\mathrm{VDW}} + \Delta \mathrm{E}_{\mathrm{elec}} + \Delta G_{\mathrm{GB}} + \Delta G_{\mathrm{SA}} \quad (2)$$

where $\Delta \mathrm{E}_{\mathrm{internal}}$ is the internal energy, $\Delta \mathrm{E}_{\mathrm{VDW}}$ is the Van Der Waals potential energy and $\Delta \mathrm{E}_{\mathrm{elec}}$ is the electrostatic potential energy, ΔG_{GB} is the polar solvation free energy and ΔG_{SA} is the nonpolar solvation free energy. Here ΔG_{GB} was calculated by generalized Born solvent model which was developed by Nguyen *et. al.* [7]ΔG_{SA} was calculated by the product of surface tension and solvent accessibility surface area, which was□$\Delta G_{\mathrm{SA}} = 0.0072 \times \Delta \mathrm{SASA}$. [8]Entropy change was ignored in this study due to high consumption of computational resources and low precision. This algorithm is implemented by gmx _MMPBSA[9].

Polyacrylamide gel electrophoresis analysis

To accurately quantify the cyclization efficiency of padlock probes with different structures, we analyzed the band intensity on the PAGE gel using Image Lab software (Version 5.2 build 14, Bio-Rad). Briefly, images of the lanes and bands on

the gel were acquired using automated scanning first. Then marked all the bands and designated the reference band. After that, relative quantification of each band was recorded in the analysis table and the average band intensity was calculated.

RCA and G-quadruplex catalytic reaction

Following cyclization, RCA reactions (20μL) were initiated by 0.6 μL (each 10 mM) dNTP Mixture, 1 μL (10U/μL) phi29 DNA polymerase (M0269L, NEB), 0.1 μL (20 ug/μL) BSA (B9200S, NEB), 0.2 μL (0.1 U/μL) IPP (M0361S, NEB) and 1X phi29 DNA Polymerase Reaction Buffer (50 mM Tris-HCl, 10 mM $MgCl_2$, 10 mM $(NH_4)_2SO_4$, 4 mM DTT, pH 7.5, at 25 °C) at 37 °C for 90 min, and inactivated at 65 °C for 10 min. Then, incubated RCA products (20 μL) with 10 μL of KCl (500 mM) and 20 μL of 1X TE at 95 °C for 10 min, then at 37 °C for 30 min. After that, 2 μL of hemin (1 mM) were added and mixed well, then continue to incubate at 37 °C for 30 min. At the same time, H_2O_2 and TMB were mixed at a ratio of 1:1 to form a chromogenic substrate. Added 50 μL of the chromogenic substrate into the above incubated solution to observe its color change using the naked eye within 10 to 30 min, recorded the optical densities at 350~700 nm at the end of incubation.

Clinical plasma samples

In total, we collected 57 EDTA-anticoagulated fresh whole blood samples, including 11 healthy individuals, 18 liver cancer, 8 lung cancer and 20 breast cancer patients, were acquired from Southwest Hospital. All blood samples were centrifuged at 1600

g for 10 min as soon as possible, then plasma was separated into fresh RNase-free tubes and frozen at -80 °C for later use. This study was approved by the Ethics Committee of First Affiliated Hospital, Army Medical University, and performed in accordance with the Declaration of Helsinki and the International Ethical Guidelines for Biomedical Research Involving Human Subjects.

Total RNA extraction

Pipette 250 μL of serum into a 2 ml tube and add 750 μL RNAiso Blood, mix well by pipetting up and down, then stand for 5 min at room temperature. Subsequently add 200 μL chloroform to the solution, shake vigorously for 15 seconds, place at rest for 5 min at room temperature and centrifuge at 12000 g for 15 min at 4 °C. Aspirate the upper aqueous phase carefully into a new tube, add
an equal volume of cold isopropanol to the aqueous solution, mix thoroughly upside down to precipitate the RNA, then put the sample at -20 °C stand for 20 min and centrifuge at 12000 g for 10 min at 4 °C. Remove the supernatant and wash the RNA pellet with an equal volume of 75% ethanol once, centrifuge at 7500 g for another 10 min at 4 °C, then discard the ethanol after the centrifugation. The RNA pellet was air-dried for 20 min and dissolved in DNase/RNase-free water. The concentration was determined by the NanoDrop One microvolume UV-Vis spectrophotometer (Thermo Fisher).

Reverse transcription PCR (RT-PCR)

The stem-loop RT-PCR was used as a standard method to analyze the concentration of miRNAs. The miRNA First Strand cDNA Synthesis Kit was used to prepare the complementary DNA (cDNA). For a 20 μL reverse transcription reaction system, mix with 1.5 μL miRNA L-RT enzyme mix, 10 μL 2X miRNA L-RT solution mix, 1 μL 10 μM primer and 3 μL total RNA solution in a 200 μL PCR tube on ice, then incubated at 16 °C for 30 min, 37 °C for 30 min, deactivated at 85 °C for 5 min and stored at 4 °C. For a 20 μL PCR system, mix with 2 μL DNF buffer, 10 μL 2X SG fast qPCR master mix, 0.4 μL 10 μM forward and reverse primers, 6 μL cDNA product and 1.2 μL H_2O in a 200 μL PCR tube on ice, then transferred to a CFX96 Real-Time System and run PCR according to the manufacturer manual: 1) activation at 95 °C for 3 min; 2) denaturation at 95 °C for 3 s, annealing at 60 °C for 30 s, 40 cycles; 3) measure melting curve from 65 °C to 95 °C with 0.5 °C increment.

Supplementary Tables

Table S1. Sequences of oligonucleotides used for miR-10b

Sequence name	Sequence (5′ to 3′) **	Length
NSLP	P-GGTTCTACAGGGTACACTTTCCTTGGTTCAGCCCCAT CCCTCCCATCCCCTTCTTCTTACATGCACAAATTC	72
SSLP-A4	P-GGTTCTACAGGGTACACTTTCCTTGGTTCAGCCCCAT CCCTCCCATCCCCTTCTTGAATCATGCACAAATTC	72
SSLP-B7	P-GGTTCTACAGGGTACAGAATAGAACCTTCATCCCCAT CCCTCCCATCCCCTTCTTCTTACATGCACAAATTC	72
SSLP-B8	P-GGTTCTACAGGGTACAGAGTAGAACCTTCATCCCCAT CCCTCCCATCCCCTTCTTCTTACATGCACAAATTC	72
DSLP-A4B5	P-GGTTCTACAGGGTACAGAATCGAACCTTCATCCCCAT CCCTCCCATCCCCTTCTTGAATCATGCACAAATTC	72
DSLP-A4B6	P-GGTTCTACAGGGTACAGAACAGAACCTTCATCCCCAT CCCTCCCATCCCCTTCTTGAATCATGCACAAATTC	72
DSLP-A4B7	P-GGTTCTACAGGGTACAGAATAGAACCTTCATCCCCAT CCCTCCCATCCCCTTCTTGAATCATGCACAAATTC	72
DSLP-A4B8	P-GGTTCTACAGGGTACAGAGTAGAACCTTCATCCCCAT CCCTCCCATCCCCTTCTTGAATCATGCACAAATTC	72
DSLP-A4B9	P-GGTTCTACAGGGTAAAGTGTAGAACCTTCATCCCCAT CCCTCCCATCCCCTTCTTGAATCATGCACAAATTC	72
DSLP-A5B8	P-GGTTCTACAGGGTACAGAGTAGAACCTTCATCCCCAT CCCTCCCATCCCCTTCTTGAATTATGCACAAATTC	72
DSLP-A6B8	P-GGTTCTACAGGGTACAGAGTAGAACCTTCATCCCCAT CCCTCCCATCCCCTTCTTGAATTTTGCACAAATTC	72
NSLP (89 nt)	P-GGTTCTACAGGGTAAATAATCCAACATTCACTCTTGCTCC TTTTTCTTCCCCATCCCTCCCATCCCCTTCTTCATTCATGCA CAAATTC	89
SSLP-A4 (89 nt)	P-GGTTCTACAGGGTAAATAATCCAACATTCACTCTTGCTCC TTTTTCTTCCCCATCCCTCCCATCCCCTTCTTGAATCATGC ACAAATTC	89
SSLP-B8 (89 nt)	P-GGTTCTACAGGGTACATAGTAGAACCTTCACTCTTGCTCC TTTTTCTTCCCCATCCCTCCCATCCCCTTCTTCATTCATGCA CAAATTC	89
DSLP-A4B5 (89 nt)	P-GGTTCTACAGGGTACATAATCGAACCTTCACTCTTGCTCCT TTTTCTTCCCCATCCCTCCCATCCCCTTCTTGAATCATGCA CAAATTC	89
DSLP-A4B6 (89 nt)	P-GGTTCTACAGGGTACATAACAGAACCTTCACTCTTGCTCC TTTTTCTTCCCCATCCCTCCCATCCCCTTCTTGAATCATGC ACAAATTC	89
DSLP-A4B7 (89 nt)	P-GGTTCTACAGGGTACAGAATAGAACCTTCACTCTTGCTCC	89

	TTTTTCTTCCCCATCCCTCCCATCCCCTTCTTGAATCATGC ACAAATTC	
DSLP-A4B8 (89 nt)	P-GGTTCTACAGGGTACATAGTAGAACCTTCACTCTTGCTCC TTTTTCTTCCCCATCCCTCCCATCCCCTTCTTGAATCATGC ACAAATTC	89
DSLP-A4B9 (89 nt)	P-GGTTCTACAGGGTAGATTGTAGAACCTTCACTCTTGCTCCT TTTTCTTCCCCATCCCTCCCATCCCCTTCTTGAATCATGCA CAAATTC	89
NSLP (62 nt)	P-GGTTCTACAGGGTAAATAATCCAACATCCCATCCCTCCCA TCCCCCATTCATGCACAAATTC	62
SSLP-A4 (62 nt)	P-GGTTCTACAGGGTAAATAATCCAACATCCCATCCCTCCCA TCCCCGAATCATGCACAAATTC	62
SSLP-B8 (62 nt)	P-GGTTCTACAGGGTACATAGTAGAACCTCCCATCCCTCCCA TCCCCCATTCATGCACAAATTC	62
DSLP-A4B5 (62 nt)	P-GGTTCTACAGGGTACATAATTGAACCTCCCATCCCTCCCA TCCCCGAATCATGCACAAATTC	62
DSLP-A4B6 (62 nt)	P-GGTTCTACAGGGTACATAACAGAACCTCCCATCCCTCCCA TCCCCGAATCATGCACAAATTC	62
DSLP-A4B7 (62 nt)	P-GGTTCTACAGGGTACAGAATAGAACCTCCCATCCCTCCCA TCCCCGAATCATGCACAAATTC	62
DSLP-A4B8 (62 nt)	P-GGTTCTACAGGGTACATAGTAGAACCTCCCATCCCTCCCA TCCCCGAATCATGCACAAATTC	62
DSLP-A4B9 (62 nt)	P-GGTTCTACAGGGTAGATTGTAGAACCTCCCATCCCTCCCA TCCCCGAATCATGCACAAATTC	62
miR-10b	UACCCUGUAGAACC/GAAUUUGUG	23
5′-SM-T *	TACCCTGTAGAACGGAATTTGTG	23
5′-SM-M1*	TACCCTGTAGTACCGAATTTGTG	23
5′-SM-M2*	AACGCTGTAGAACCGAATTTGTG	23
3′-SM-T *	TACCCTGTAGAACCCAATTTGTG	23
3′-SM-M1*	TACCCTGTAGAACCGATTTTGTG	23
3′-SM-M2*	TACCCTGTAGAACCGAATTTATG	23
DM-8/11*	TACCCTGTAGAAGCGTATTTGTG	23
SM-5	UACCCUGUAGAACCGAAUAUGUG	23
SM-9	UACCCUGUAGAACCCAAUUUGUG	23
DM-8/11	UACCCUGUAGAAGCGUAUUUGUG	23
DM-5/10	UACCCUGUAGAACGGAAUAUGUG	23
DM-9/10	UACCCUGUAGAACGCAAUUUGUG	23
TM-8/11/13	UACCCUGUAGUAGCGUAUUUGUG	23
TM-5/9/19	UACCGUGUAGAACCCAAUAUGUG	23

10b-Specific primer	TACCCTGTAGAACCGAATTTGTG	23
Universal primer	CCCATCCCTCCCATCCCC	18
10b-PCR-RT	GTCGTATCCAGTGCAGGGTCCGAGGTATTCGCACTGGATACGACCACAAA	50
10b-PCR-F	AACGATATACCCTGTAGAACCGA	23
10b-PCR-R	CAGTGCAGGGTCCGAGGT	18

* Just for NUPACK simulation. ** 'P' in the sequence represented the phosphate group.

Table S2. Sequences of oligonucleotides used for miR-155

Sequence name	Sequence (5′ to 3′) *	Length
NSLP	P-ACGATTAGCATTAACACATACCAACCTTCACTCCCAT CCCTCCCATCCCCTTCTTGAATCATGACCCCTATC	72
SSLP-A4	P-ACGATTAGCATTAACACATACCAACCTTCACTCCCAT CCCTCCCATCCCCTTCTTGATACATGACCCCTATC	72
SSLP-B8	P-ACGATTAGCATTAACACACTAATCGTTTCACTCCCAT CCCTCCCATCCCCTTCTTGAATCATGACCCCTATC	72
DSLP-A4B5	P-ACGATTAGCATTAACAGATAGATCGTTTCACTCCCAT CCCTCCCATCCCCTTCTTGATACATGACCCCTATC	72
DSLP-A4B6	P-ACGATTAGCATTAACAGATAAATCGTTTCACTCCCAT CCCTCCCATCCCCTTCTTGATACATGACCCCTATC	72
DSLP-A4B7	P-ACGATTAGCATTAACAGATTAATCGTTTCACTCCCAT CCCTCCCATCCCCTTCTTGATACATGACCCCTATC	72
DSLP-A4B8	P-ACGATTAGCATTAACAGACTAATCGTTTCACTCCCAT CCCTCCCATCCCCTTCTTGATACATGACCCCTATC	72
DSLP-A4B9	P-ACGATTAGCATTAACTGGCTAATCGTTTCACTCCCAT CCCTCCCATCCCCTTCTTGATACATGACCCCTATC	72
miR-155	UUAAUGCUAAUCGUGAUAGGGGU	23
155-SM	UUAAUGCUAAUCGUCAUAGGGGU	23
155-Specific primer	TTAATGCTAATCGTGATAGGGGT	23
155-PCR-RT	GTCGTATCCAGTGCAGGGTCCGAGGTATTCGCACTGGATA CGACACCCCT	50
155-PCR-F	AAGCGCCTTTAATGCTAATCGT	22
155-PCR-R	CAGTGCAGGGTCCGAGGT	18

* ‘P’ in the sequence represented the phosphate group.

Table S3. Sequences of oligonucleotides used for miR-21

Sequence name	Sequence (5′ to 3′) *	Length
NSLP	P-GTCTGATAAGCTAGTCTATAGAAACCTTCACTCCCAT CCCTCCCATCCCCTTCTACAATCTACTCAACATCA	72
SSLP-A5	P-GTCTGATAAGCTAGTCTATAGAAACCTTCACTCCCAT CCCTCCCATCCCCTTCTTGATGAATCTCAACATCA	72
SSLP-B8	P-GTCTGATAAGCTACAACATATCAGACTTCACTCCCAT CCCTCCCATCCCCTTCTACAATCTACTCAACATCA	72
DSLP-A5B5	P-GTCTGATAAGCTACAAGAATACAGACTTCACTCCCAT CCCTCCCATCCCCTTCTTGATGAATCTCAACATCA	72
DSLP-A5B6	P-GTCTGATAAGCTACAAGAATTCAGACTTCACTCCCAT CCCTCCCATCCCCTTCTTGATGAATCTCAACATCA	72
DSLP-A5B7	P-GTCTGATAAGCTACAAGAAATCAGACTTCACTCCCAT CCCTCCCATCCCCTTCTTGATGAATCTCAACATCA	72
DSLP-A5B8	P-GTCTGATAAGCTACAACATATCAGACTTCACTCCCAT CCCTCCCATCCCCTTCTTGATGAATCTCAACATCA	72
DSLP-A5B9	P-GTCTGATAAGCTACAAGTTATCAGACTTCACTCCCAT CCCTCCCATCCCCTTCTTGATGAATCTCAACATCA	72
miR-21	UAGCUUAUCAGACUGAUGUUGA	22
21-SM	UAGCUUAUCAGAGUGAUGUUGA	22
21-Specific primer	TAGCTTATCAGACTGATGTTGA	22
21-PCR-RT	GTCGTATCCAGTGCAGGGTCCGAGGTATTCGCACTGGATA CGACTCAACA	50
21-PCR-F	AAGAGCGTTAGCTTATCAGACTG	23
21-PCR-R	CAGTGCAGGGTCCGAGGT	18

* ‘P’ in the sequence represented the phosphate group.

Table S4. Sequences of oligonucleotides used for miR-192

Sequence name	Sequence (5′ to 3′) *	Length
NSLP	P-ATTCATAGGTCAGTCGCCATACGTAGCATCACTCCCAT CCCTCCCATCCCCATCTTGAATCTTAGGCTGTCA	72
SSLP-A4	P-ATTCATAGGTCAGTCGCCATACGTAGCATCACTCCCAT CCCTCCCATCCCCATCTTGACTCTTAGGCTGTCA	72
SSLP-B8	P-ATTCATAGGTCAGTCGCGCTATGAATCATCACTCCCAT CCCTCCCATCCCCATCTTCACTCTTAGGCTGTCA	72
DSLP-A4B5	P-ATTCATAGGTCAGTCGCGAATTGAATCATCACTCCCAT CCCTCCCATCCCCATCTTGACTCTTAGGCTGTCA	72
DSLP-A4B6	P-ATTCATAGGTCAGTCGCGAAATGAATCATCACTCCCAT CCCTCCCATCCCCATCTTGACTCTTAGGCTGTCA	72
DSLP-A4B7	P-ATTCATAGGTCAGTCGCGATATGAATCATCACTCCCAT CCCTCCCATCCCCATCTTGACTCTTAGGCTGTCA	72
DSLP-A4B8	P-ATTCATAGGTCAGTCGCGCTATGAATCATCACTCCCAT CCCTCCCATCCCCATCTTGACTCTTAGGCTGTCA	72
DSLP-A4B9	P-ATTCATAGGTCAGTCGCCCTATGAATCATCACTCCCAT CCCTCCCATCCCCATCTTGACTCTTAGGCTGTCA	72
miR-192	CUGACCUAUGAAUUGACAGCC	21
192-SM	CUGACCUAUGAAUAGACAGCC	21
192-Specific primer	CTGACCTATGAATTGACAGCC	21

* ‘P’ in the sequence represented the phosphate group.

Table S5. Sequences of oligonucleotides used for miR-26a

Sequence name	Sequence (5′ to 3′) *	Length
NSLP	P-TGGATTACTTGAACACATACGAACCATTAACTCCCAT CCCTCCCATCCCCTTCTTGAATCAACAGCCTATCC	72
SSLP-A4	P-TGGATTACTTGAACACATACGAACCATTAACTCCCAT CCCTCCCATCCCCTTCTTGGATCAACAGCCTATCC	72
SSLP-B8	P-TGGATTACTTGAACAGATGTAATCCATTAACTCCCAT CCCTCCCATCCCCTTCTTGAATCAACAGCCTATCC	72
DSLP-A4B5	P-TGGATTACTTGAACACATACGATCCATTAACTCCCAT CCCTCCCATCCCCTTCTTGGATCAACAGCCTATCC	72
DSLP-A4B6	P-TGGATTACTTGAACACATACAATCCATTAACTCCCAT CCCTCCCATCCCCTTCTTGGATCAACAGCCTATCC	72
DSLP-A4B7	P-TGGATTACTTGAACAGATATAATCCATTAACTCCCAT CCCTCCCATCCCCTTCTTGGATCAACAGCCTATCC	72
DSLP-A4B8	P-TGGATTACTTGAACAGATGTAATCCATTAACTCCCAT CCCTCCCATCCCCTTCTTGGATCAACAGCCTATCC	72
DSLP-A4B9	P-TGGATTACTTGAACAGTAGTAATCCATTAACTCCCAT CCCTCCCATCCCCTTCTTGGATCAACAGCCTATCC	72
miR-26a	UUCAAGUAAUCCAGGAUAGGCU	22
26a-SM	UUCAAGUAAUCCACGAUAGGCU	22
26a-Specific primer	TTCAAGTAATCCAGGATAGGCT	22

* ‘P’ in the sequence represented the phosphate group.

Table S6. Sequences of oligonucleotides used for DENV-2

Sequence name	Sequence (5′ to 3′) *	Length
NSLP (DENV-2)	P-CTTCTGAATCTCTTGTTGTTATCTTCGCCCATCCCTCC CATCCCCATTTTTTTTTTTTTTTTTTTTTATCGTTGCATAT CCTCTTCC	87
SSLP-A5 (DENV-2)	P-CTTCTGAATCTCTTGTTGTTATCTTCGCCCATCCCTCC CATCCCCATTTTTTTTTTTTTTTTTTTTTATGGAAGCATAT CCTCTTCC	87
SSLP-B8 (DENV-2)	P-CTTCTGAATCTCTTGTTCTTCAGAAGTCCCATCCCTC CCATCCCCATTTTTTTTTTTTTTTTTTTTTATCGTTGCAAA TCCTCTTCC	87
DSLP-A5B5 (DENV-2)	P-CTTCTGAATCTCTTGTTGTATAGAAGGCCCATCCCTC CCATCCCCATTTTTTTTTTTTTTTTTTTTTACGGAAGCATA TCCTCTTCC	87
DSLP-A5B6 (DENV-2)	P-CTTCTGAATCTCTTGTTCAACAGAAGGCCCATCCCTC CCATCCCCATTTTTTTTTTTTTTTTTTTTTACGGAAGCATA TCCTCTTCC	87
DSLP-A5B7 (DENV-2)	P-CTTCTGAATCTCTTGTTCATCAGAAGTCCCATCCCTC CCATCCCCATTTTTTTTTTTTTTTTTTTTTACGGAAGCATA TCCTCTTCC	87
DSLP-A5B8 (DENV-2)	P-CTTCTGAATCTCTTGTTCTTCAGAAGTCCCATCCCTC CCATCCCCATTTTTTTTTTTTTTTTTTTTTACGGAAGCATA TCCTCTTCC	87
DSLP-A5B9 (DENV-2)	P-CTTCTGAATCTCTTGTTATTCAGAAGTCCCATCCCTC CCATCCCCATTTTTTTTTTTTTTTTTTTTTACGGAAGCATA TCCTCTTCC	87
DENV-2	ACUACAUGCCAUCCAUGAAGAGAUUCAGAAGGGAAGA GGAAGAGGCAGGUGUCCUGUGGU	60
DENV-2-SM	ACUACAUGCCAUCCAUGAAGAGAUUCAGAAGAGAAGA GGAAGAGGCAGGUGUCCUGUGGU	60
DENV-2-SP	ACAAGAGATTCAGAAGGGAAG	21

* ‘P’ in the sequence represented the phosphate group.

Table S7. Comparison of recent studies on the detection of miRNAs

Method	Target	Output Method	Linear Range	LOD	Highest multiplex	Ref.
PECL-CRISPR	miR-17	ECL	1 fM-100 pM	1 fM	1	10
HRCA-CRISPR	miR-17	FL	0.5 fM-50 fM	200 aM	1	11
TSDR	miR-21	SERS/ EC	0.5 fM-0.5 pM /5 fM-100 pM	0.12 fM /2.2 fM	1	12
MNAzyme	miR-21/miR-155 /miR-10b	ICP-MS	50 pM-1 nM/50 pM-2 nM /50 pM-2 nM	10-20 pM	3	13
EDC	miR-21/miR-155 /miR-10b	ICP-MS	20 pM-1 nM	7.4 pM/7.5 pM /11 pM	3	14
CHA	miR-21	PEC/electrochromic	100 fM-10 μM /100 fM-1 μM	58.0 fM /48.5 fM	1	15
DSNA-HAC	miR-21	FL	25 pM-5 nM	17 pM	1	16
HCR	miR-21	Phosphorescence/ ECL	5 pM-2 nM /1 pM-0.8 nM	1.40 pM /0.18 pM	1	17
HRCA	miR-10b/miR-21 /miR-155/miR-26a /miR-192; DENV-2	FL	0.5 fM–5 nM	133.9 aM	Not less than 3	this work

Supplementary Figures

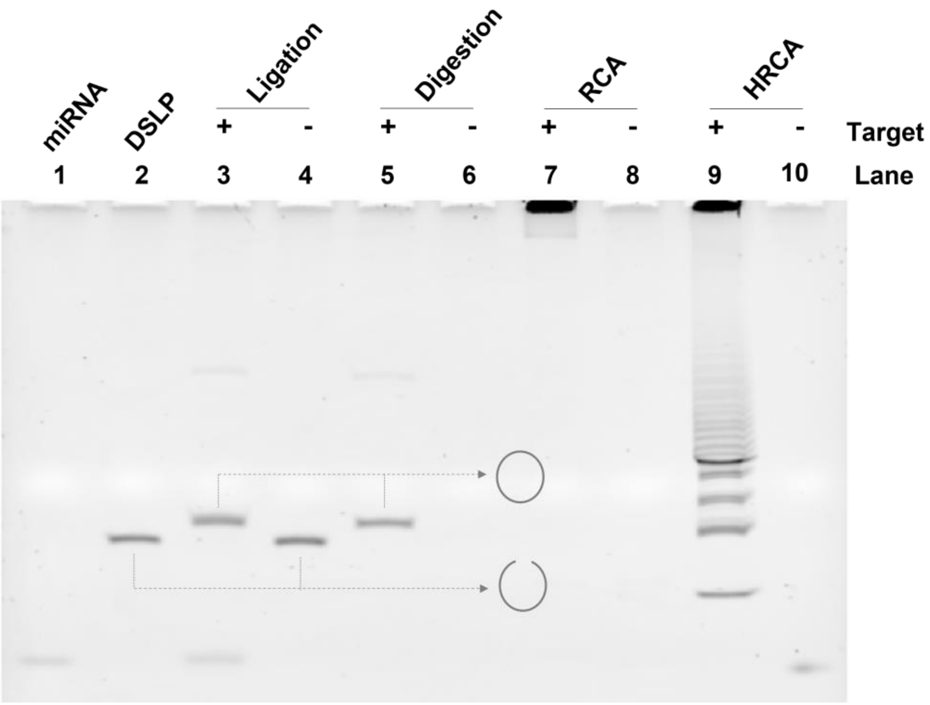

Figure S1. Denatured PAGE for the process of DSLP reaction. Lane 1: target miR-10b; Lane 2: DSLP probe; Lane 3-4: ligation products with and without target; Lane 5-6: Digestion products of Lane 3 and 4 using exonuclease I and III. Since linear oligonucleotides were digested, no band was found in lane 6; Lane 7-8: RCA products with and without target; Lane 9-10: HRCA products with and without target.

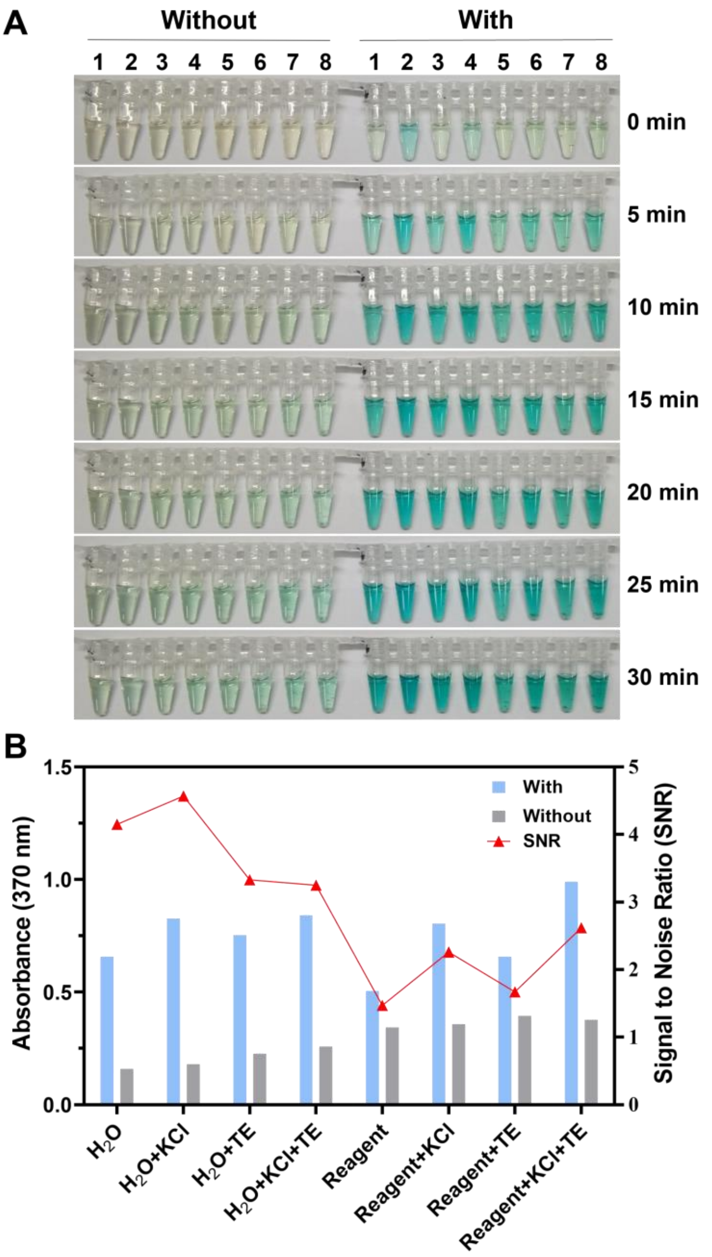

Figure S2. Feasibility verification and condition exploration of G-quadruplex catalytic property. **(A)** Catalytic effect under different conditions with and without G-quadruplex sequence (complementary sequence of functional region). Number 1 to 8 represent the following eight catalytical conditions: only H2O, H2O + KCl, H2O + TE, H2O + KCl + TE, only reagent of RCA reaction, RCA reagent + KCl, RCA reagent + TE, RCA reagent + KCl + TE. After adding the TMB chromogenic substrate, incubated at 37 °C and started timing, recorded the graphic photos at 0min, 5min, 10min, 15min, 20min, 25min and 30min, and

measured the absorbance at 370 nm after 30min. **(B)** The absorbance of each sample at 370 nm after incubation for 30min and the signal-to-noise ratio (SNR, with-to-without ratio) under each condition.

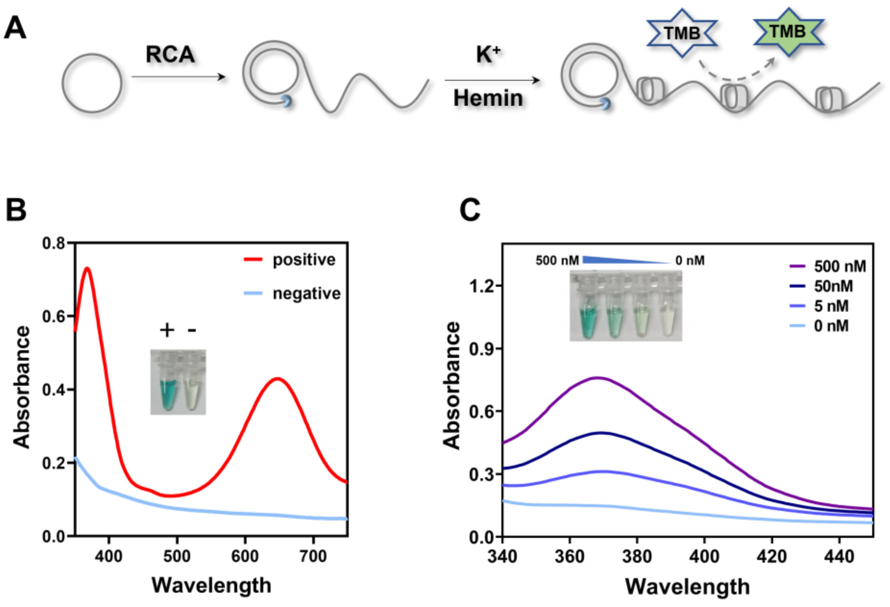

Figure S3. The validation for the catalytical efficacy of the functional region of DSLP as the G-quadruplex. (A) Schematic of TMB oxidation reaction based on RCA. **(B)** The absorption spectra of the TMB oxidation reaction based on RCA, C_{miRNA} = 500 nM. With the presence of target, the absorption peaks of TMB at 370 nm and 652 nm were both appeared with a 4.4-fold and 7.5-fold increasement respectively. **(C)** The graphic image and the absorption spectra at 370 nm of the TMB oxidation reaction based on the RCA reaction with different concentrations of target (miR-10b), 2.1-fold change for 5nM, 3.3-fold change for 50nM, 5.1-fold change for 500nM, respectively.

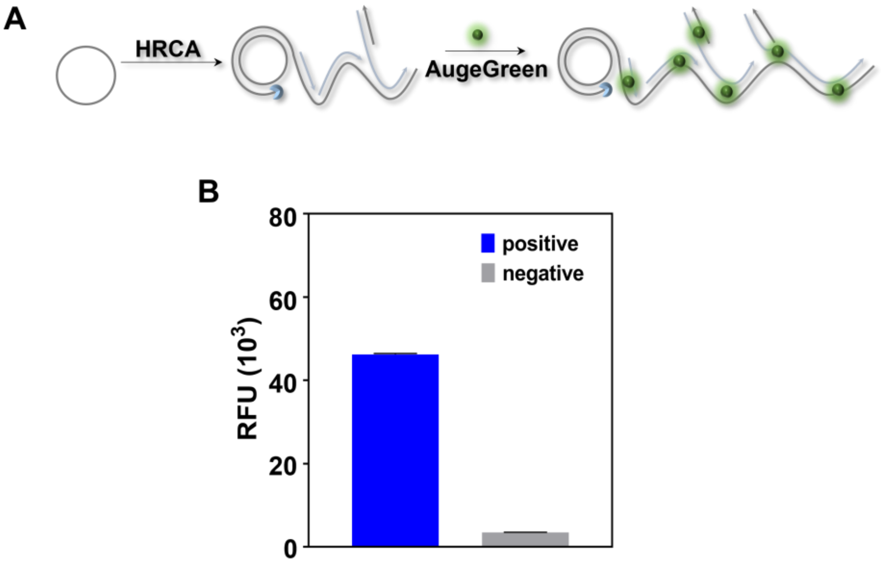

Figure S4. The validation of the efficacy of functional region of DSLP as a HRCA primer site. (A) Schematic of Fluorescence enhancement based on HRCA. **(B)** The relative fluorescence unit (RFU) of HRCA base on DSLP with and without target, C_{miRNA} = 500 nM.

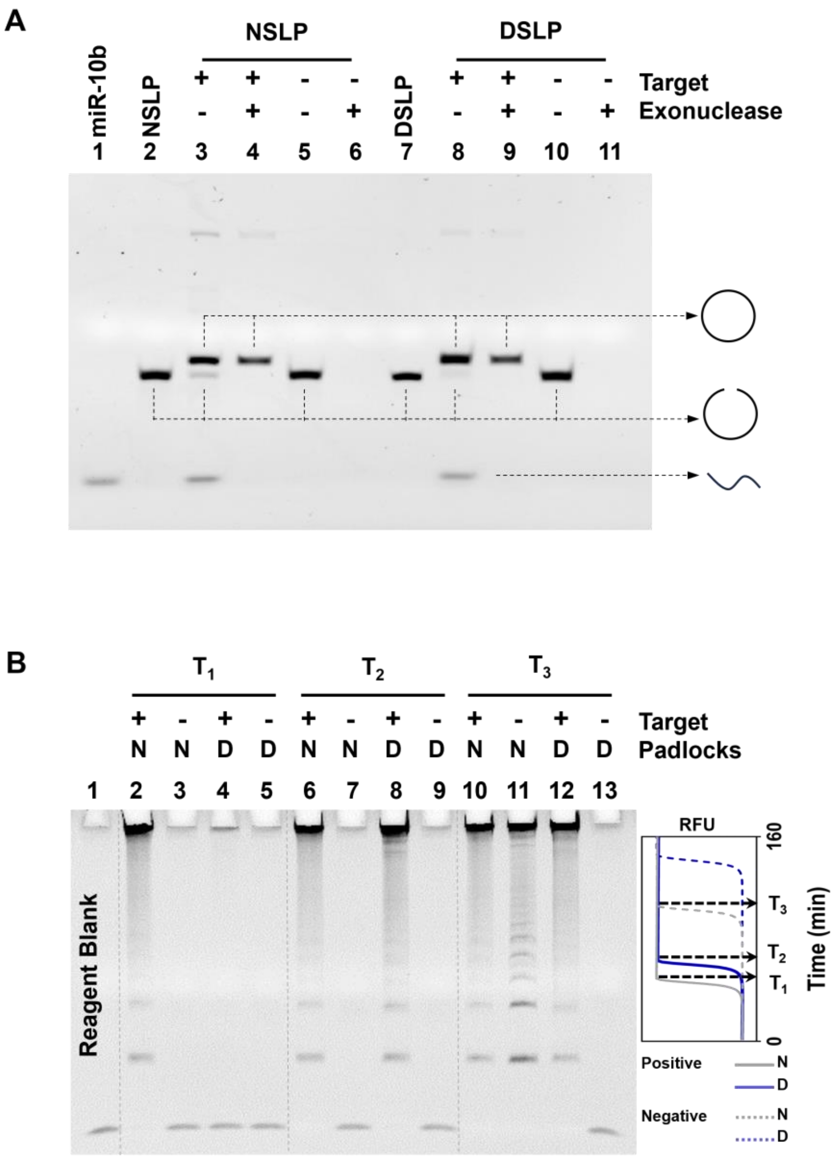

Figure S5. Comparison of cyclization reactions and HRCA reactions for the NSLP and the DSLP. (A) Comparison of cyclization reactions for the NSLP and the DSLP. C_{padlock} = 500 nM, C_{miRNA} = 500 nM, CPBCV-1 DNA ligase = 1.25 U/μL, 1X buffer, 25°C, 3 h. (B) Comparison of HRCA reactions for the NSLP and the DSLP. T_1, T_2 and T_3 stand for three distinct time points at which we collect the reaction samples. N and D stand for NSLP and DSLP, respectively.

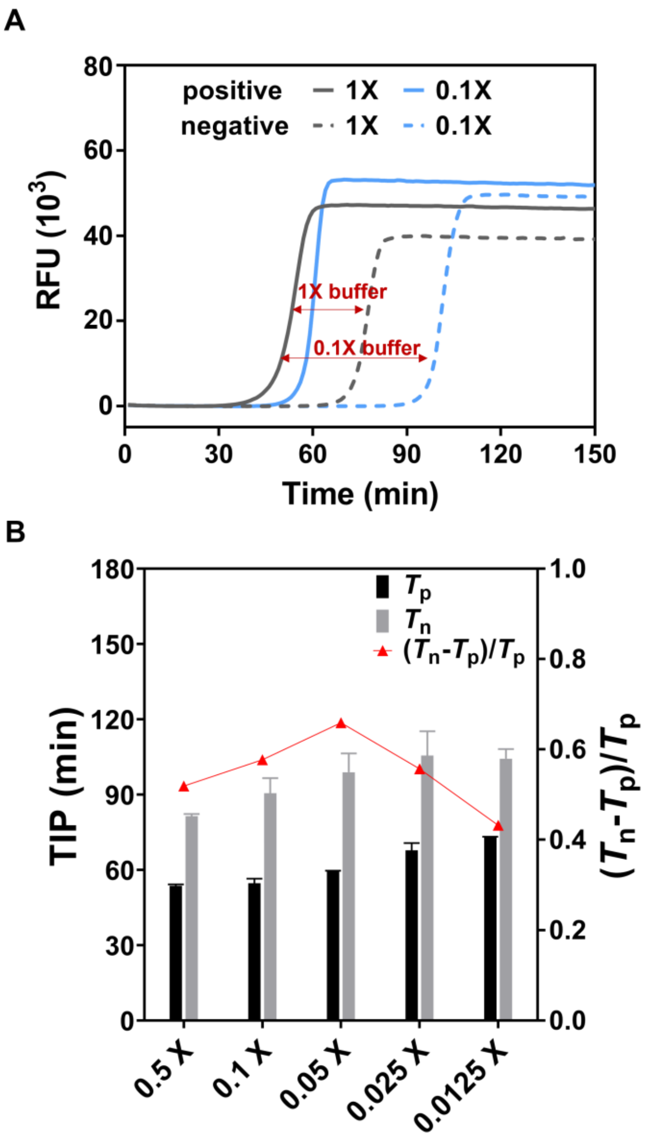

Figure S6. The effect of different ligation buffer concentration. (A) Preliminary exploration of the effect of ligation buffer concentration on detection signal. The reduced buffer concentration suppressed the negative signal and was beneficial to SNR. **(B)** Systematic study of the effect of different ligation buffer concentrations on $(T_n\text{-}T_p)/T_p$ (T_p and T_n were the times of inflection point with and without the target). C_{miRNA} = 500 nM, Mean ± SD, n = 3. The best SNR was obtained when the ligase buffer was 0.05X.

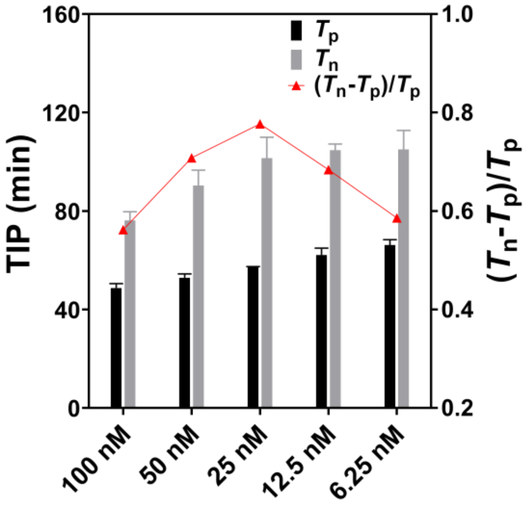

Figure S7. The effect of different concentration of padlock probe. Systematic study of the effect of different concentration of padlock probe on $(T_n$-$T_p)/T_p$ (T_p and T_n were the times of inflection point with and without the target). C_{miRNA} = 500 nM, Mean ± SD, n = 3. The best SNR was obtained when the concentration of padlock probe was 25 nM.

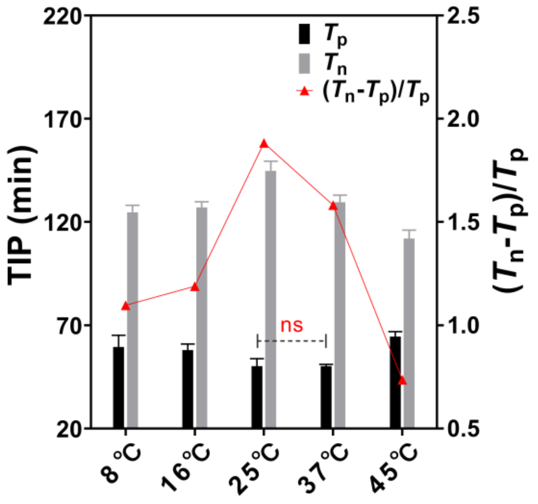

Figure S8. The effect of different cyclization temperatures. Systematic study of the effect of different cyclization temperatures on $(T_n-T_p)/T_p$ (T_p and T_n were the times of inflection point with and without the target). C_{miRNA} = 500 nM, Mean ± SD, n = 3. The best SNR was obtained when the cyclization temperature was 25 °C. (one-way ANOVA and Dunnett's multiple comparison test, ns denotes $p > 0.05$). Analysis of the data from positive samples reveals that 25°C and 37°C exhibit similar times of inflection point (TIP), both of which are superior to higher and lower temperatures. This observation is likely attributed to the temperature-dependent impact on DNA ligase activity. When examining the data from negative samples, it was evident that the suppression of the negative signal by DSLP decrease progressively with increasing temperature. This could be attributed to the fact that the stability of the secondary structure being influenced at higher temperatures.

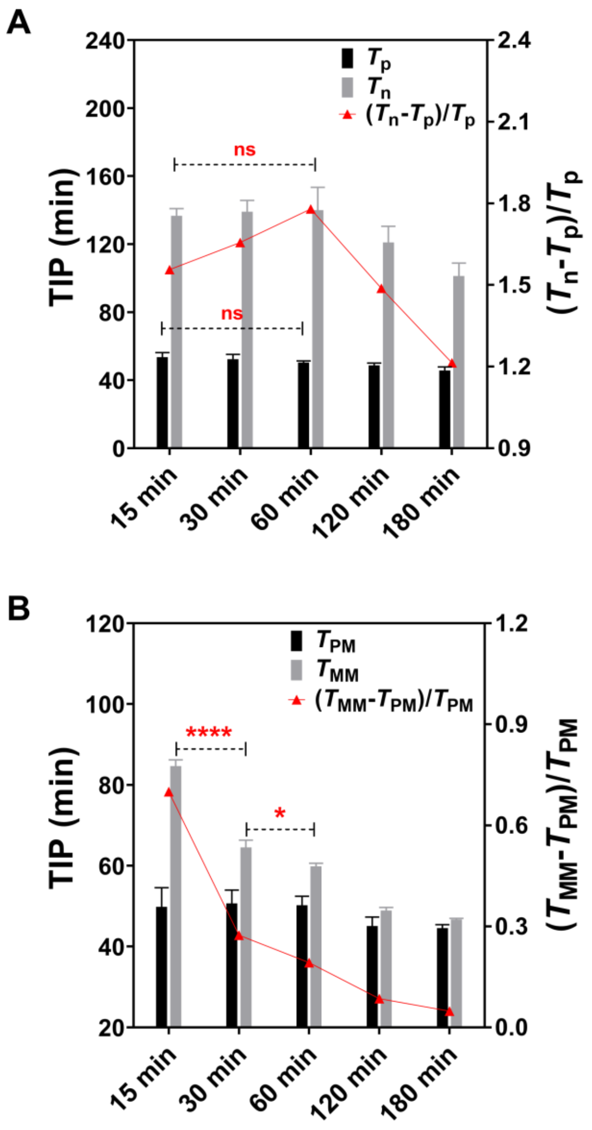

Figure S9. The effect of different reaction times on signal-to-noise ratio and mismatch discrimination. (A) Systematic study of the effect of different reaction times on $(T_n\text{-}T_p)/T_p$ (T_p and T_n were the times of inflection point with and without the target). C_{miRNA} = 500 nM, Mean ± SD, n = 3. The best reaction time seemed to have obtained at 60 min, but there was no statistically significant difference between negative reaction times within 60 min, and the same for positive reaction times, suggesting that 15min, 30min and 60min had similar SNRs (one-way analysis of variance (ANOVA), ns denotes $p > 0.05$). **(B)** Systematic study of the effect of different reaction times on $(T_{MM}\text{-}T_{PM})/T_{PM}$ (T_{MM} and T_{PM} were inflection point time

with mismatched or perfect matched sequence). C_{miRNA} = 500 nM, Mean ± SD, n = 3. The mismatch discrimination was significantly better at 15 min than at longer times, making 15 min the optimum response time (one-way ANOVA and Tukey's multiple comparison test, * denotes $P < 0.05$, **** denotes $P < 0.0001$).

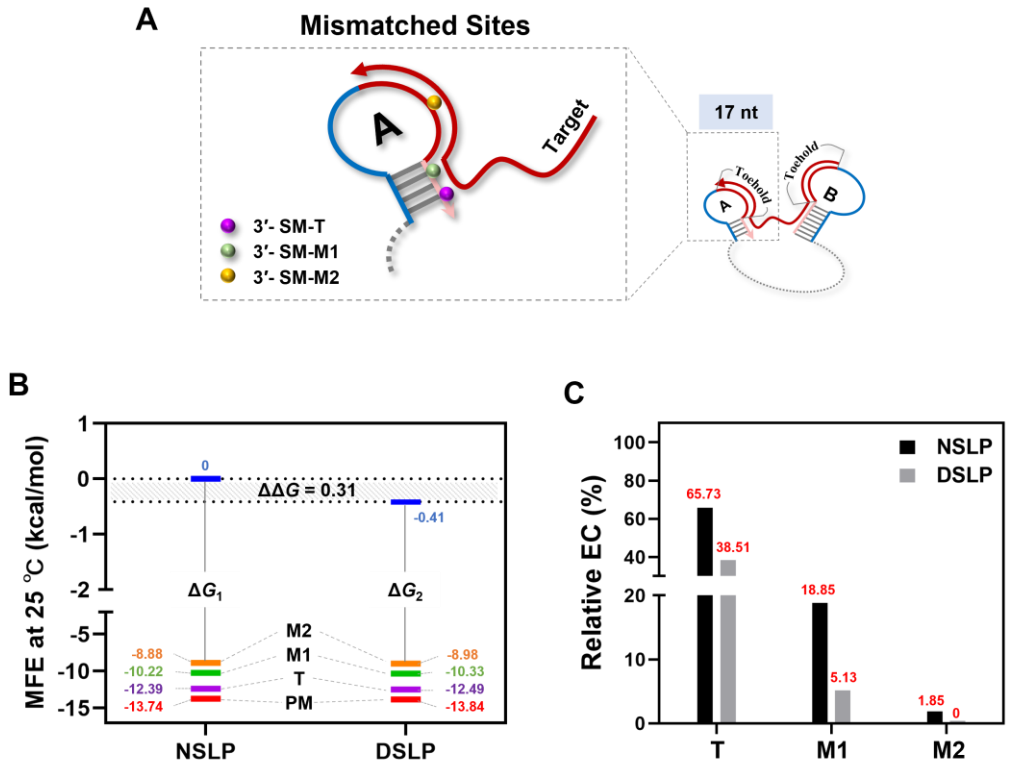

Figure S10. NUPACK simulation for the binding state of the SL-A region to mismatched targets. (A) Schematic of the mismatched sites between the mismatched target and the SL-A region. 3′-SM-T (SM: single-base mismatch): with a mismatched site at the 3′ terminal of the SL-A, 3′-SM-M1: with a mismatched site in the middle of the SL-A stem, 3′-MM-M2: with a mismatched site in the middle of the complementary segment of the target and the SL-A loop. **(B)** Minimum free energy (MFE) of the SL-A region of NSLP and DSLP before and after binding to different targets at 25 °C. MFE difference of the SL-A region before and after binding to target for NSLP and DSLP was ΔG_1 and ΔG_2, respectively. $\Delta\Delta G = \Delta G_1 - \Delta G_2$, mainly from the energy difference in the initial conformation between NSLP and DSLP. **(C)** Equilibrium concentration (EC) of the SL-A region of NSLP and DSLP combined with different mismatched targets. Using the EC that binding to PM as 1, the relative EC that binding to mismatched target was calculated. The simulation indicated that whichever

position the mismatched site was located, DSLP had a lower relative EC than NSLP. PM: perfect matched target; T, M1 and M2 represent 3′-SM-T, 3′-SM-M1 and 3′-MM-M2 respectively in figures B and C.

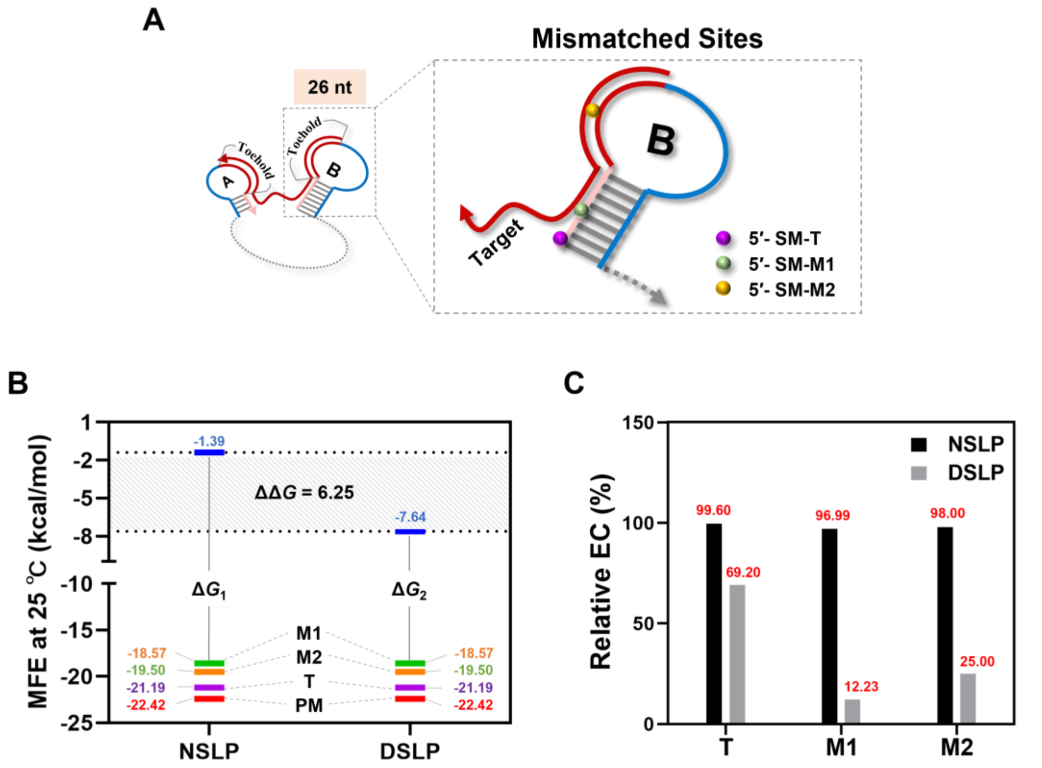

Figure S11. NUPACK simulation for the binding state of the SL-B region to mismatched targets. (A) Schematic of the mismatched sites between the mismatched target and the SL-B region. 5′-SM-T: with a mismatched site at the 5′ terminal of the SL-B, 5′-SM-M1: with a mismatched site in the middle of the SL-B stem, 5′-SM-M2: with a mismatched site in the middle of the complementary segment of the target and the SL-B loop. **(B)** Minimum free energy (MFE) of the SL-B region of NSLP and DSLP before and after binding to different targets at 25 °C. MFE difference of the SL-B region before and after binding to target for NSLP and DSLP was ΔG_1 and ΔG_2, respectively. $\Delta\Delta G = \Delta G_1 - \Delta G_2$, mainly from the energy difference in the initial conformation between NSLP and DSLP. **(C)** Equilibrium concentration (EC) of the SL-B region of NSLP and DSLP combined with different mismatched targets. Using the EC binding to PM as 1, the relative EC binding to mismatched target was calculated. The simulation indicated that whichever position the mismatched site

was located, DSLP had a lower relative EC than NSLP. PM: perfect matched target; T, M1 and M2 represent 3′-SM-T, 3′-SM-M1 and 3′-MM-M2 respectively in figures B and C.

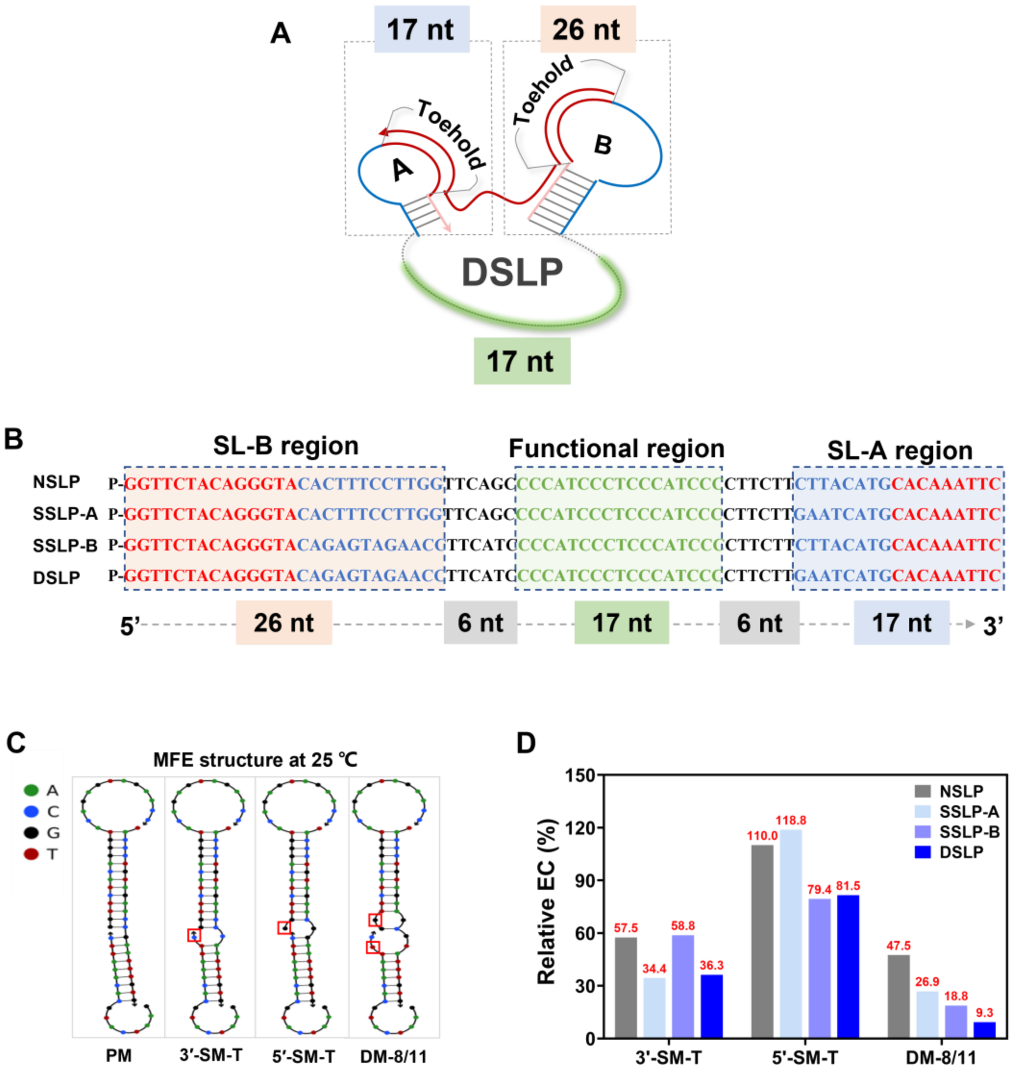

Figure S12. NUPACK simulation for the simultaneous binding state of the SL-A and SL-B regions to mismatched targets. **(A)** Schematic diagram of the DSLP structure and **(B)** the sequences of four different structural padlock probes, showing specific details of the different probe structures. **(C)** The minimum free energy (MFE) structures of SL-B region and SL-A region simultaneously bound to different targets. **(D)** Equilibrium concentrations (EC) of the SL-A and SL-B regions of four padlock probes binding simultaneously to different mismatched targets. Using the EC binding to PM as 1, the relative EC binding to mismatched targets was calculated. PM: perfect matched target; 3′-SM-T: a target with a mismatched site at the 3′ terminal of SL-A, 5′-SM-T: a target with a mismatched site at 5′ terminal of SL-B.

DM-8/11: a double-base mismatched target with two mismatched sites next to both ends of the nick (mismatches were located at the 8th and 11th bases from the 3′ terminal of the target).

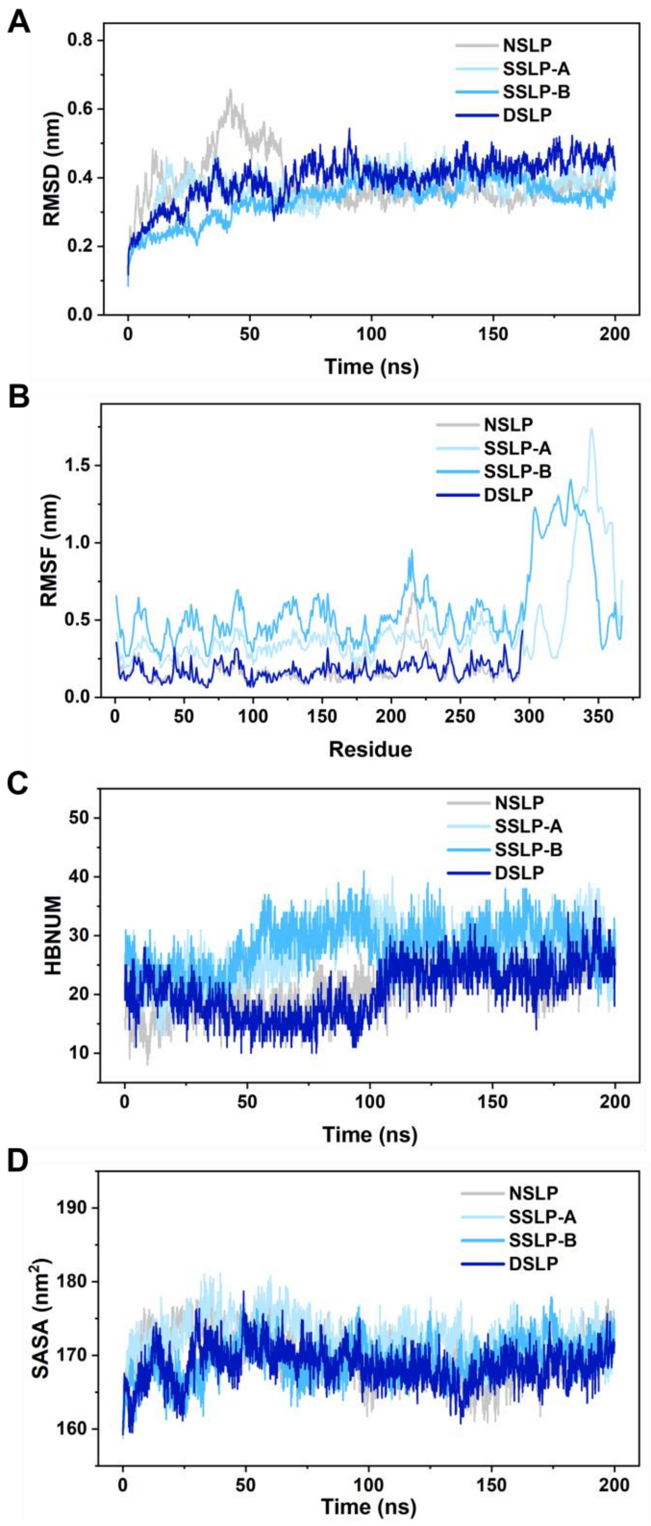

Figure S13. ~200 ns molecular dynamics (MD) simulation of padlocks with four structures. (A) The root mean square deviation (RMSD) of MD simulation. **(B)** The root mean square function (RMSF) of MD simulation. **(C)** The hygrogen bond number (HBNUM) of MD simulation. **(D)** The solvent accessible surface area (SASA) of MD simulation.

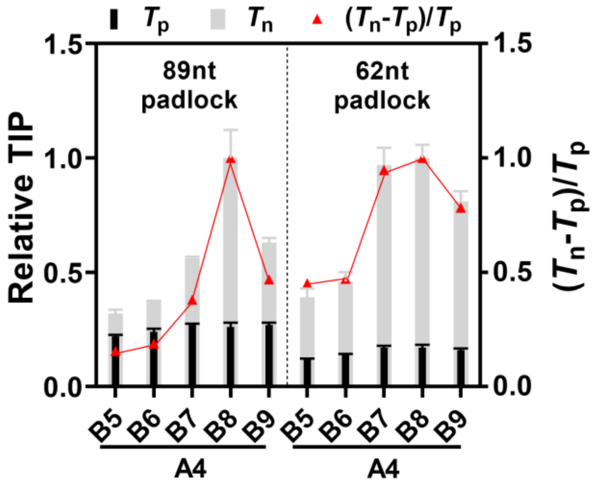

Figure S14. Optimization of the B-stem length for 89 nt and 62 nt DSLP probes. Keeping the A-stem at 4bp and changing the B-stem from 5bp to 9bp, the times of inflection point (TIP) of the amplification curves of different B-stem length with and without target were recorded (T_p and T_n were the times of inflection point with and without the target). C_{miRNA} = 500 nM, mean ± SD, n = 3.

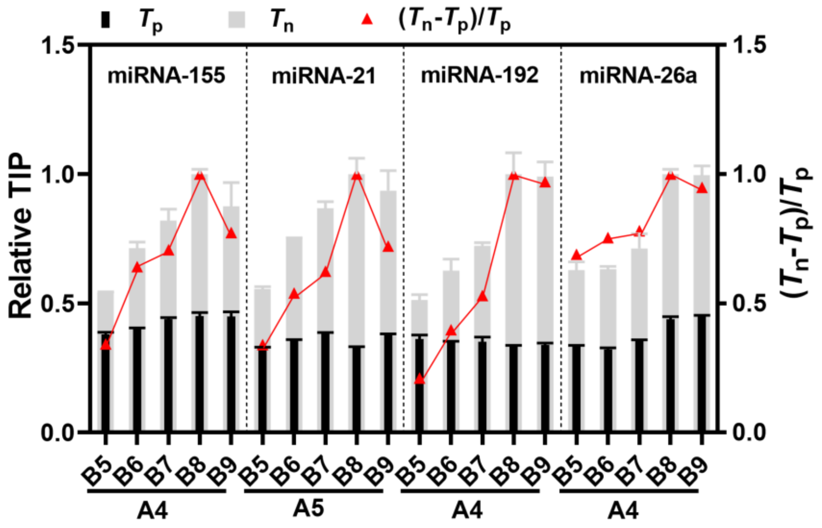

Figure S15. The B-stem length optimization of four DSLP probes targeting different miRNAs. Keeping the A-stem at 4bp or 5bp and changing the B-stem from 5bp to 9bp, the times of inflection point (TIP) of the amplification curves of different B-stem length with and without target were recorded (T_p and T_n were the times of inflection point with and without the target). C_{miRNA} = 500 nM, mean ± SD, n = 3.

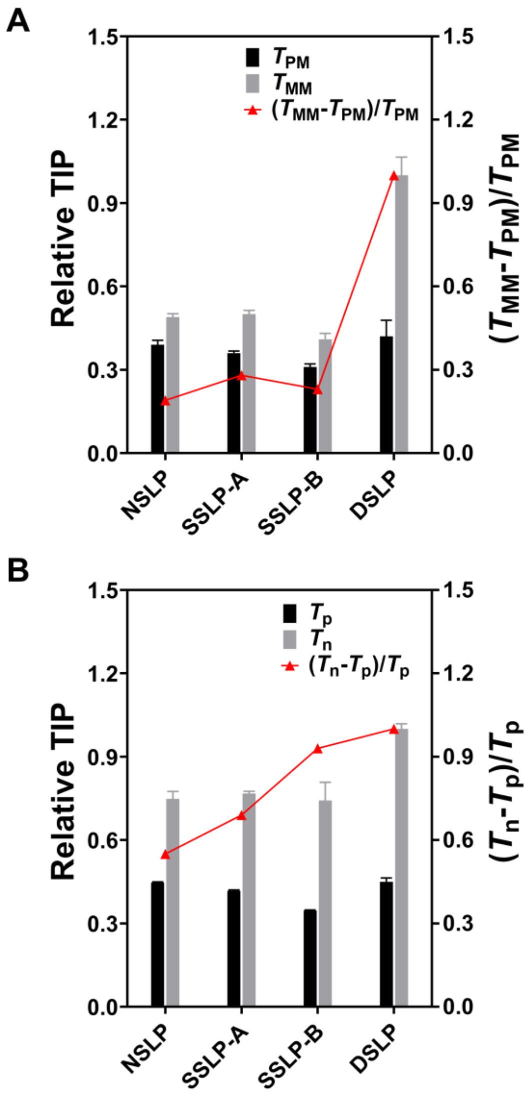

Figure S16. Comparison of mismatch recognition and signal-to-noise ratios (SNRs) for different structural padlocks for miRNA-155 detection. (A) Comparison of mismatch recognition for different structural padlocks. $(T_{MM}-T_{PM})/T_{PM}$ was used to evaluate the mismatch recognition ability of different structural probes (T_{MM} and T_{PM} were the times of inflection point (TIP) with and without mismatched target). C_{miRNA} = 500 nM, Mean ± SD, n = 3. The results clearly showed that the DSLP was significantly better than other structural padlock probes. **(B)** Comparison of SNRs for different structural padlocks. $(T_n-T_p)/T_p$ was used to evaluate SNRs of different structural probes (T_p and T_n were the times of inflection point (TIP) with and without target). C_{miRNA} = 500 nM, Mean ± SD, n = 3. The SNR increased

with the total number of complementary bases of the stem-loops, and the DSLP obtained the best SNR.

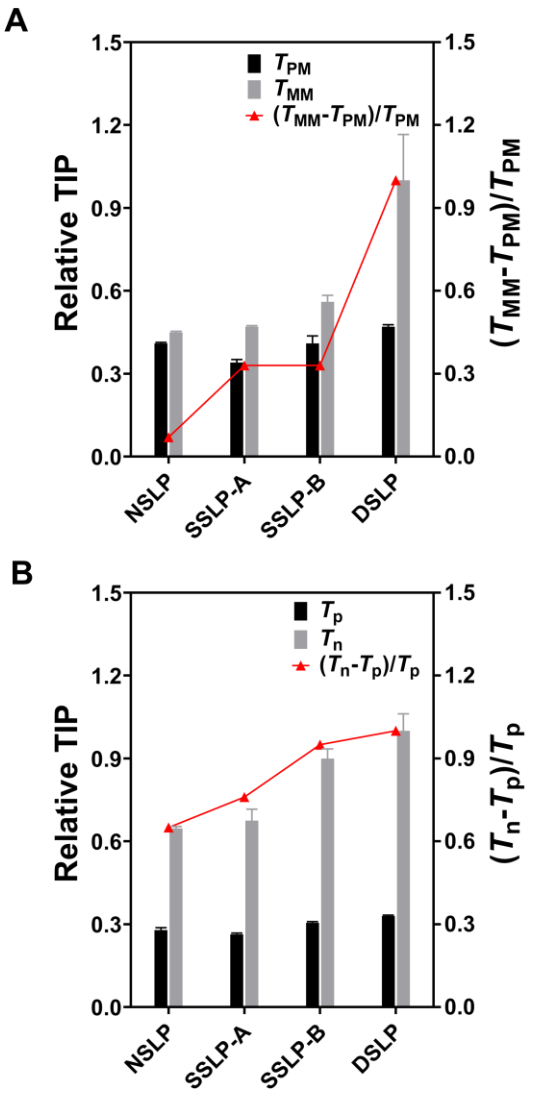

Figure S17. Comparison of mismatch recognition and signal-to-noise ratios (SNRs) for different structural padlocks for miRNA-21 detection. (A) Comparison of mismatch recognition for different structural padlocks. $(T_{MM}-T_{PM})/T_{PM}$ was used to evaluate the mismatch recognition ability of different structural probes (T_{MM} and T_{PM} were the times of inflection point (TIP) with and without mismatched target). C_{miRNA} = 500 nM, Mean ± SD, n = 3. The results clearly showed that the DSLP was significantly better than other structural padlock probes, while the NSLP was the worst. **(B)** Comparison of SNRs for different structural padlocks. $(T_n-T_p)/T_p$ was used to evaluate SNRs of different structural probes (T_p and T_n were the times of inflection point with and without target). C_{miRNA} = 500 nM, Mean ±

SD, n = 3. The SNR increased with the total number of complementary bases of the stem-loops, and the DSLP obtained the best SNR.

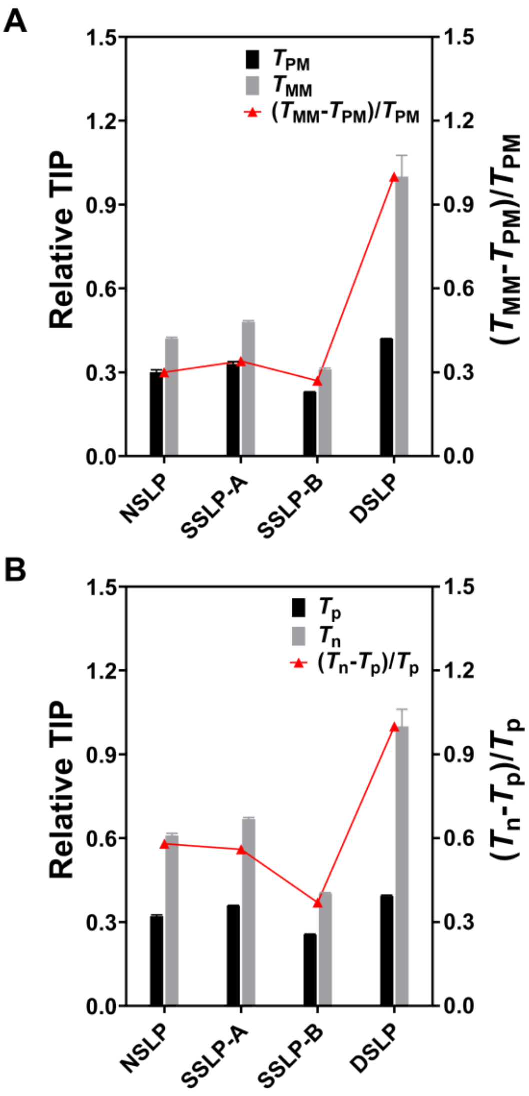

Figure S18. Comparison of mismatch recognition and signal-to-noise ratios (SNRs) for different structural padlocks for miRNA-192 detection. (A) Comparison of mismatch recognition for different structural padlocks. $(T_{MM}-T_{PM})/T_{PM}$ was used to evaluate the mismatch recognition ability of different structural probes (T_{MM} and T_{PM} were the times of inflection point with and without mismatched target). C_{miRNA} = 500 nM, Mean ± SD, n = 3. The results clearly showed that the DSLP was significantly better than other structural padlock probes. **(B)** Comparison of SNRs for different structural padlocks. $(T_n-T_p)/T_p$ was used to evaluate SNRs of different structural probes (T_p and T_n were the times of inflection point with and without target). C_{miRNA} = 500 nM, Mean ± SD, n = 3. The SNRs didn't

increase with the total number of complementary bases of the stem-loops, SSLP-B surprisingly appeared the lowest SNR. However, the DSLP still achieved the best SNR.

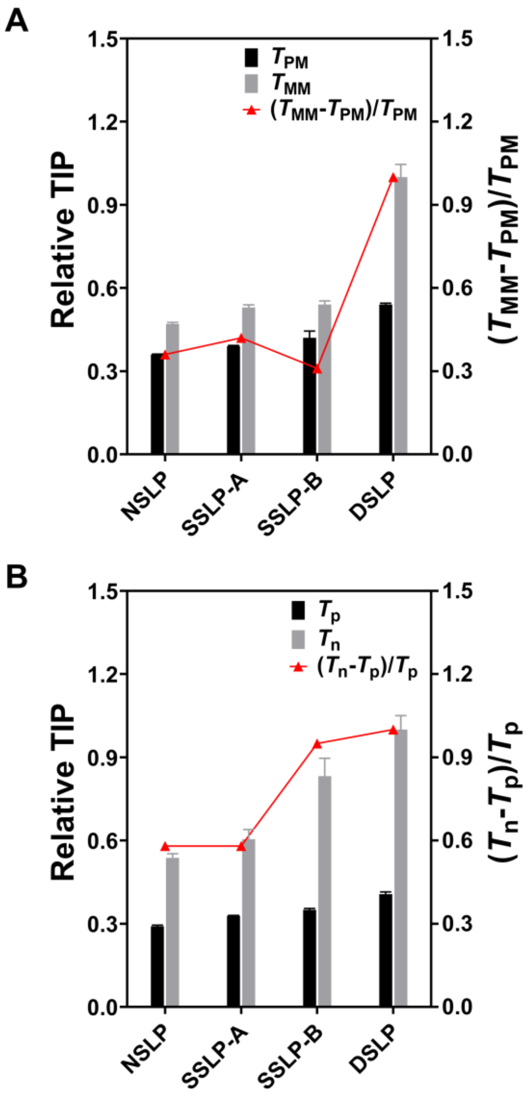

Figure S19. Comparison of mismatch recognition and signal-to-noise ratios (SNRs) for different structural padlocks for miRNA-26a detection. (A) Comparison of mismatch recognition for different structural padlocks. $(T_{MM}-T_{PM})/T_{PM}$ was used to evaluate the mismatch recognition ability of different structural probes (T_{MM} and T_{PM} were the times of inflection point with and without mismatched target). C_{miRNA} = 500 nM, Mean ± SD, n = 3. The results clearly showed that the DSLP was significantly better than other structural padlock probes. **(B)** Comparison of SNRs for different structural padlocks. $(T_n-T_p)/T_p$ was used to evaluate SNRs of different structural probes (T_p and T_n were the times of inflection point with and without target). C_{miRNA} = 500 nM, Mean ± SD, n = 3. The SNR increased with

the total number of complementary bases of the stem-loops, and the DSLP obtained the best SNR.

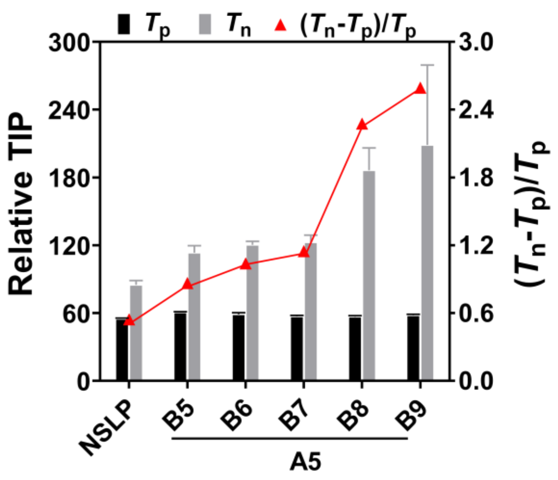

Figure S20. The B-stem length optimization of DSLP targeting DENV-2. Keeping the A-stem of DSLP at 5bp and changing the B-stem from 5bp to 9bp, the times of inflection point (TIP) of the amplification curves of different structural padlock probes with and without target were recorded (T_p and T_n were the times of inflection point with and without the target). C_{miRNA} = 500 nM, mean ± SD, n = 3.

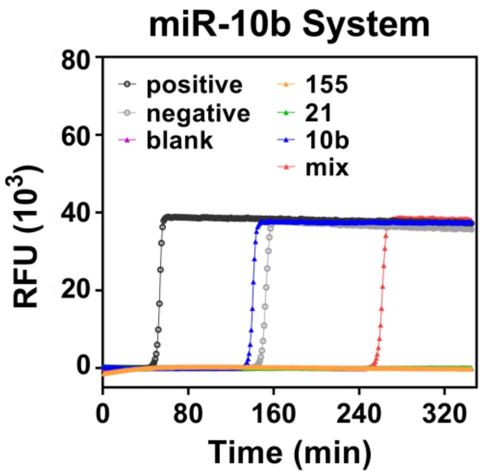

Figure S21. Amplification curves of miR-10b HRCA systems. Positive and negative: cyclization products of DSLP probe targeting miR-10b in the presence and absence of target, C_{miRNA} = 500 nM; blank: HRCA reaction system of miR-10; 155, 21 and 10b: DSLP probes targeting miR-155, miR-21 and miR-10b respectively, at 6-fold the experimental concentration; mix: DSLP probes targeting miR-155, miR-21 and miR-10b were mixed at twice the experimental concentration. Comparing the "10b" sample (blue curve) with the "mix" sample (red curve), both containing only uncyclized DSLP but at different concentrations (6-fold the experimental concentration for "10b" and 2-fold for "mix"), the amplification signals were delayed from approximately 150 minutes to about 250 minutes with the decrease in DSLP concentration, demonstrating that the uncyclized DSLP is one of the sources of the background signal. Given that the signal intensity of uncyclized DSLP at twice the experimental concentration was already much lower than that of negative samples (including non-specific cyclized products and uncyclized DSLP), we conclude that the background signal resulting from uncyclized DSLP is negligible when compared to the signal arising from non-specific cyclized DSLP.

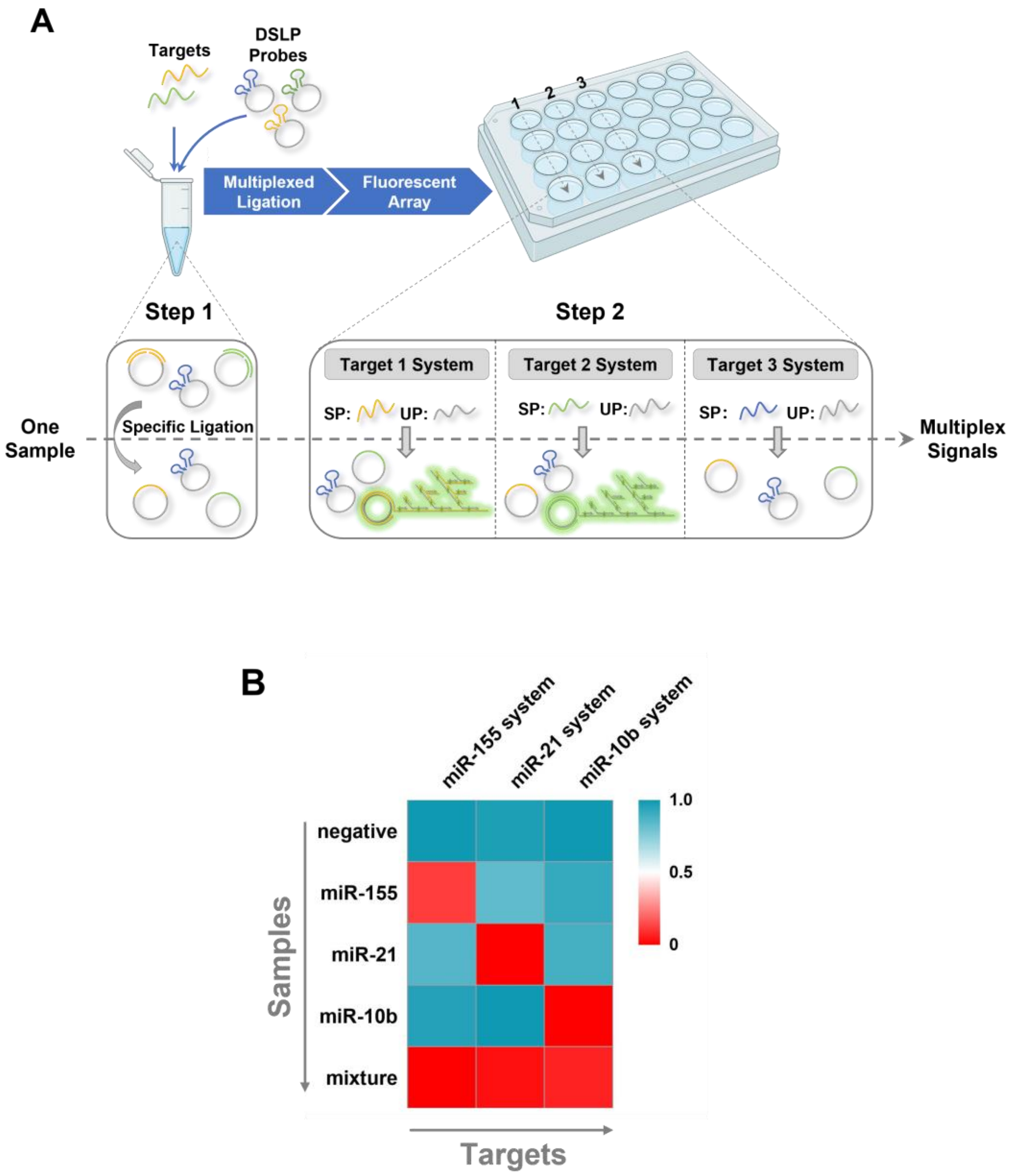

Figure S22. Feasibility verification of multiplex detection based on DSLP probes. (A) Schematic diagram of the multiplex detection principle. The process involved two steps: specific multiplex ligation (step 1) and HRCA-based fluorescent microarrays (step 2). Three specific DSLP probes were premixed and specifically cyclized in a one-pot reaction when their respective target was present. The cyclization products were then added to the HRCA-based fluorescent microarrays. Each column of the microarray contained only one specific primer (SP) that recognized the cyclized chain associated with the respective target, triggered the HRCA reaction, and output a fluorescent signal (as shown in the Target 1 and 2 system). If

the target corresponding to the SP did not exist, its specific DSLP probe could not be cyclized and the HRCA could not be performed. In this case, there was no fluorescence signal output (as shown in the Target 3 system). **(B)** Response of different targets in fluorescent microarrays. Samples containing different targets were prepared to verify the feasibility of multiplex detection using the procedure described above. We normalized the fluorescence signal (TIP) of each column (i.e., one of miRNA systems) to the interval [0, 1]. As expected, only the sample containing specific targets can generate positive signal in the respective HRCA array, and when other non-specific targets were present, fluorescent signals were comparable to negative signals.

Supplementary Reference

(1) Zadeh, J. N.; Steenberg, C. D.; Bois, J. S.; Wolfe, B. R.; Pierce, M. B.; Khan, A. R.; Dirks, R. M.; Pierce, N. A. NUPACK: Analysis and design of nucleic acid systems. *J. Comput. Chem.* **2011**, *32* (1), 170-173.

(2) Lopez, R.; Chen, Y.; Dumas Ang, S.; Yekhanin, S.; Makarychev, K.; Racz, M.; Seelig, G.; Strauss, K.; Ceze, L. DNA assembly for nanopore data storage readout. *Nat. Commun.* **2019**, *10* (1), 2933.

(3) Xiong, Y.; Huang, Q.; Canady, T.; Barya, P.; Liu, S.; Arogundade, O.; Race, C.; Che, C.; Wang, X.; Zhou, L.; Wang, X.; Kohli, M.; Smith, A.; Cunningham, B. Photonic crystal enhanced fluorescence emission and blinking suppression for single quantum dot digital resolution biosensing. *Nat. Commun.* **2022**, *13* (1), 4647.

(4) Yan, Y.; Tao, H.; He, J.; Huang, S. Y. The HDOCK server for integrated protein-protein docking. *Nat. Protoc.* **2020**, *15* (5), 1829-1852.

(5) Pettersen, E. F.; Goddard, T. D.; Huang, C. C.; Meng, E. C.; Couch, G. S.; Croll, T. I.; Morris, J. H.; Ferrin, T. E. UCSF ChimeraX: Structure visualization for researchers, educators, and developers. *Protein Sci.* **2021**, *30* (1), 70-82.

(6) Van Der Spoel, D.; Lindahl, E.; Hess, B.; Groenhof, G.; Mark, A. E.; Berendsen, H. J. GROMACS: fast, flexible, and free. *J. Comput. Chem.* **2005**, *26* (16), 1701-1718.

(7) Nguyen, H.; Roe, D. R.; Simmerling, C. Improved Generalized Born Solvent Model Parameters for Protein Simulations. *J. Chem. Theory Comput.* **2013**, *9* (4), 2020-2034.

(8) Weiser, J.; Shenkin, P. S.; Still, W. C. Approximate atomic surfaces from linear combinations of pairwise overlaps (LCPO). *J. Comput. Chem.* **1999**, *20* (2), 217-230.

(9) Valdés-Tresanco, M.; Valdés-Tresanco, M.; Valiente, P.; Moreno, E. gmx_MMPBSA: A New Tool to Perform End-State Free Energy Calculations with GROMACS. *J. Chem. Theory Comput.* **2021**, *17* (10), 6281-6291.

(10) Zhou, T.; Huang, R.; Huang, M.; Shen, J.; Shan, Y.; Xing, D. CRISPR/Cas13a Powered Portable Electrochemiluminescence Chip for Ultrasensitive and Specific MiRNA Detection. *Adv. Sci.* **2020**, *7* (13), 1903661.

(11) Huang, M.; Huang, R.; Yue, H.; Shan, Y.; Xing, D. Ultrasensitive and high-specific microRNA detection using hyper-branching rolling circle amplified CRISPR/Cas13a biosensor. *Sens. Actuators, B* **2020**, *325*, 128799.

(12) Zhou, H.; Zhang, J.; Li, B.; Liu, J.; Xu, J.-J.; Chen, H.-Y. Dual-Mode SERS and Electrochemical Detection of miRNA Based on Popcorn-like Gold Nanofilms and Toehold-Mediated Strand Displacement Amplification Reaction. *Anal. Chem.* **2021**, *93* (15), 6120-6127.

(13) Kang, Q.; He, M.; Chen, B.; Xiao, G.; Hu, B. MNAzyme-Catalyzed Amplification Assay with Lanthanide Tags for the Simultaneous Detection of Multiple microRNAs by Inductively Coupled Plasma–Mass Spectrometry. *Anal. Chem.* **2020**, *93* (2), 737-744.

(14) Kang, Q.; Chen, B.; He, M.; Hu, B. Simple Amplifier Coupled with a Lanthanide Labeling Strategy for Multiplexed and Specific Quantification of MicroRNAs. *Anal. Chem.* **2022**, *94* (37), 12934-12941.

(15) Li, M.; Liu, Z.; Liu, Y.; Luo, H.; Huang, K.-J.; Tan, X. Capacitor-parallel-amplified decoupled photoelectrochemical/electrochromic dual-mode bioassay for sensitive detection of microRNA with high reliability. *Biosens. Bioelectron.* **2023**, *232*, 115310.

(16) Duan, L.-Y.; Hong, Y.-P.; Yang, W.-Y.; Zhang, L.-L.; Liu, J.-W. Hairpin cascade circuits on programmable DNA-nucleated spherical nucleic acid for intracellular targeted microRNA imaging. *Chem. Eng. J.* **2023**, *473*.

(17) Li, H.; Yang, Q.; Wang, Z.; Li, F. Iridium Complex with Specific Intercalation in the G-Quadruplex: A Phosphorescence and Electrochemiluminescence Dual-Mode Homogeneous Biosensor for Enzyme-Free and Label-Free Detection of MicroRNA. *ACS Sens.* **2023**, *8* (4), 1529-1535.
